# Host restriction of *Escherichia coli* recurrent urinary tract infection occurs in a bacterial strain-specific manner

**DOI:** 10.1371/journal.ppat.1007457

**Published:** 2018-12-13

**Authors:** Valerie P. O’Brien, Denise A. Dorsey, Thomas J. Hannan, Scott J. Hultgren

**Affiliations:** 1 Department of Molecular Microbiology and Center for Women’s Infectious Disease Research, Washington University School of Medicine, St. Louis, Missouri, United States of America; 2 Department of Pathology and Immunology, Washington University School of Medicine, St. Louis, Missouri, United States of America; University of Toronto, CANADA

## Abstract

Urinary tract infections (UTI) are extremely common and can be highly recurrent, with 1–2% of women suffering from six or more recurrent episodes per year. The high incidence of recurrent UTI, including recurrent infections caused by the same bacterial strain that caused the first infection, suggests that at least some women do not mount a protective adaptive immune response to UTI. Here we observed in a mouse model of cystitis (bladder infection) that infection with two different clinical uropathogenic *Escherichia coli* (UPEC) isolates, UTI89 or CFT073, resulted in different kinetics of bacterial clearance and different susceptibility to same-strain recurrent infection. UTI89 and CFT073 both caused infections that persisted for at least two weeks in similar proportions of mice, but whereas UTI89 infections could persist indefinitely, CFT073 infections began to clear two weeks after inoculation and were uniformly cleared within eight weeks. Mice with a history of CFT073 cystitis lasting four weeks were protected against recurrent CFT073 infection after antibiotic therapy, but were not protected against challenge with UTI89. In contrast, mice with a history of UTI89 cystitis lasting four weeks were highly susceptible to challenge infection with either strain after antibiotic treatment. We found that depletion of CD4^+^ and CD8^+^ T cell subsets impaired the ability of the host to clear CFT073 infections and rendered mice with a history of CFT073 cystitis lasting four weeks susceptible to recurrent CFT073 cystitis upon challenge. Our findings demonstrate the complex interplay between the broad genetic diversity of UPEC and the host innate and adaptive immune responses during UTI. A better understanding of these host-pathogen interactions is urgently needed for effective drug and vaccine development in the era of increasing antibiotic resistance.

## Author summary

Many patients suffer from highly recurrent urinary tract infections (UTI) caused by *Escherichia coli*, which are genetically diverse bacteria. Recurrent episodes are often caused by the same *E*. *coli* strain that caused the first infection, suggesting that some patients may not develop a protective immune response. We found that two *E*. *coli* UTI strains behaved differently in a mouse model of UTI. One strain, UTI89, could infect the bladder indefinitely, whereas strain CFT073 was always cleared within eight weeks. After mice had a CFT073 infection and antibiotic treatment, they were protected from a CFT073 challenge infection, but were susceptible to a UTI89 challenge infection. In contrast, mice with a UTI89 infection and antibiotics were susceptible to recurrent UTI when challenged with either strain. We found that depleting T cells, immune cells important for developing protection against infection, prevented mice from clearing their CFT073 infections and made them susceptible to recurrent CFT073 UTI. Thus, while infection with one *E*. *coli* strain could trigger a protective immune response, another strain sidestepped this response. Our findings shed new light on immune responses to UTI and may be important for drug and vaccine development.

## Introduction

Urinary tract infections (UTI) are among the most common infections: more than half of women, as well as some men, will have at least one UTI in their lifetime [1], mostly caused by uropathogenic *E*. *coli* (UPEC) [2,3]. In humans, the adaptive immune response to UTI, and particularly to cystitis (bladder infection), is not well understood. Placebo studies show that in some women an acute UTI spontaneously resolves, whereas other women develop symptomatic or asymptomatic colonization lasting weeks [4–8]. UTI can also be highly recurrent: after receiving appropriate antibiotic therapy, 20–30% of women with an acute UTI will have a recurrent episode (rUTI) within six months [1], despite the fact that about half of rUTI episodes are caused by the same UPEC strain that caused the initial infection [9]. These findings suggest that individuals with UTI frequently fail to mount an adaptive immune response that protects against subsequent infection, even with the same strain. Although bacterial factors and host responses that impact innate immune responses to UTI have been characterized [10,11], less is known about host and/or microbial factors that impact the host’s ability to mount a protective adaptive immune response.

Studies of experimental infection in different strains of inbred mice have illustrated the complexity of the role of adaptive immunity in determining UTI outcomes. In C57BL/6 mice, UPEC infection is inherently self-limiting, independent of infectious dose, and adaptive immune responses can partially protect from recurrent infection [12,13]. For example, the adoptive transfer of either cellular or humoral immunity after experimental UPEC bladder infection partially protects naive C57BL/6 mice against infection with an isogenic UPEC strain [12]. Other strains of mice have been shown to be susceptible to same-strain recurrences caused by the well-studied clinical cystitis isolate UTI89 [14,15]. For example, C3H/HeN mice can develop chronic and severe same-strain recurrent infections with UTI89 in an infectious dose-dependent manner [14], indicating that adaptive immunity towards UTI89 is insufficient to prevent these outcomes. However, vaccination of C3H mice with bacterial antigens can strongly protect against both acute and recurrent UTI [15,16], demonstrating that a protective adaptive response to UTI can be experimentally induced in these mice.

**Fig 1:**
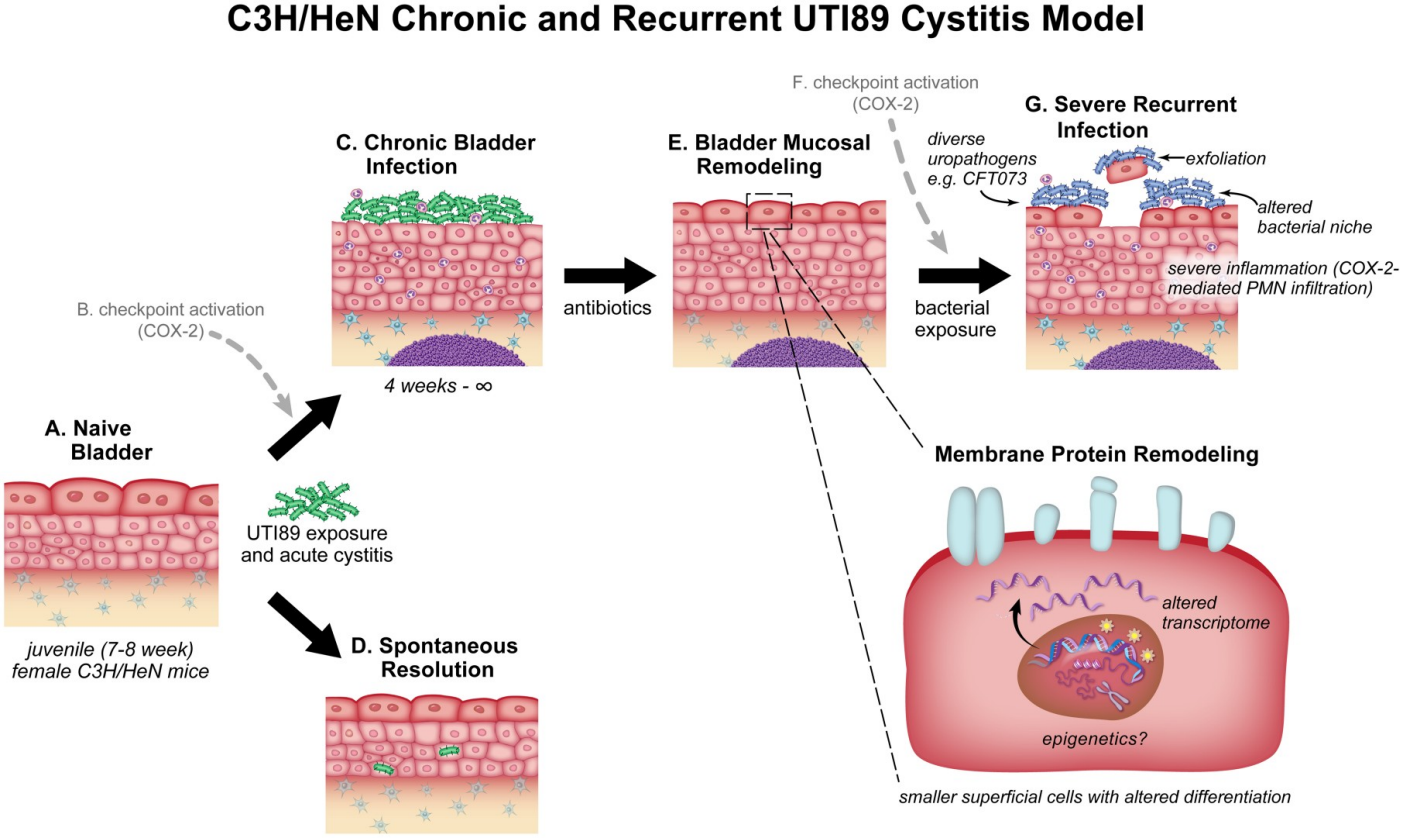
The C3H/HeN mouse model of chronic and recurrent cystitis using the clinical cystitis isolate UTI89. (**A**) In the inbred mouse strain C3H/HeN, inoculation with 10^8^ CFU of the clinical UPEC isolate UTI89 can trigger a cyclooxygenase-2 (COX-2)-dependent acute host-pathogen checkpoint (**B**) comprising high urine bacterial titers, elevated proinflammatory cytokines in the serum, polymorphonuclear neutrophils (PMNs) in urine, and severe bladder inflammation and mucosal wounding [14,17]. (**C**) Mice that trigger the checkpoint develop chronic cystitis (persistent bacteriuria ≥10^4^ CFU/ml urine coupled with bladder titers ≥10^4^ CFU and bladder inflammation at sacrifice) lasting for four weeks or more [14]; (**D**) mice that do not trigger the checkpoint spontaneously resolve the infection and develop sterile urine [14]. (**E**) In mice that develop chronic cystitis, antibiotic therapy administered at four weeks post infection sterilizes the bladder; however, we found that the bladder does not return to its naive state. Instead, the bladder harbors a “molecular imprint” of infection, comprising changes to the bladder transcriptome [15], smaller superficial cells with altered differentiation [15], a remodeled epithelial membrane proteome [17], and the presence of lymphoid follicles [14]. When “challenged” with a second bacterial exposure (typically 10^7^ CFU), these remodeled bladders respond differently from age-matched naive bladders: if cyclooxygenase-2 (COX-2)-mediated inflammation and checkpoint activation is triggered [15,17] (**F**), then (**G**) the host develops severe recurrent chronic cystitis [14], which can be caused by UTI89 or other uropathogens such as the urosepsis isolate CFT073 [15]. During infection in the remodeled bladder, the bacterial niche is altered relative to the naive bladder (intracellular replication is reduced) and the host responds with COX-2-mediated PMN infiltration and exfoliation of epithelial cells [15]. In the present study, we investigated whether infection of juvenile C3H/HeN mice with CFT073 would result in the same constellation of phenotypes seen with UTI89 infection, i.e. checkpoint activation, chronic cystitis and bladder remodeling, and susceptibility to recurrent UTI.

Our previous studies in C3H/HeN mice (summarized in Fig 1) have revealed that within 24 hours post infection (hpi) with UTI89, a cyclooxygenase-2-dependent acute host-pathogen checkpoint determines the outcome of the infection [14,17]. The hallmarks of “checkpoint activation” are high urine bacterial titers (bacteriuria), pyuria (neutrophil accumulation in urine), elevated serum levels of pro-inflammatory cytokines, and severe bladder inflammation with bladder epithelial wounding. Elements of this checkpoint are predictive of recurrent UTI in women, demonstrating clinical relevance of the model [17]. Mice that do not activate the checkpoint spontaneously resolve the infection within four weeks, and have bladder titers <10^4^ colony-forming units (CFU) at time of sacrifice [14]. In contrast, mice that activate the checkpoint develop chronic cystitis, which we define as persistent, high-titer bacteriuria (>10^4^ CFU/ml urine for four weeks) coupled with high bladder bacterial burden (>10^4^ CFU/bladder) and bladder inflammation at sacrifice four weeks post infection (wpi). Strikingly, we have found that those mice that develop chronic cystitis lasting at least four weeks remain infected apparently indefinitely (six months or longer) [14], indicating that, once established, chronic UTI89 bladder infections cannot be cleared by adaptive immunity. Antibiotics can clear the chronic infection, but we found that a “molecular imprint” remains on the bladder mucosa in the form of significant bladder mucosal remodeling [15,17] that modulates host response to challenge infection. Thus, mice with a history of chronic UTI89 cystitis are highly susceptible (or “sensitized”) to checkpoint activation and severe recurrent chronic cystitis upon challenge infection with either UTI89 or other clinical uropathogenic strains [15,17].

In this study, we used these well-characterized mouse models of chronic and recurrent UTI in C3H/HeN mice (Fig 1) to probe the roles of bacterial diversity and adaptive immunity in UTI by comparing and contrasting the disease outcomes of two clinical UPEC isolates: the cystitis isolate UTI89 [18] and the pyelonephritis/urosepsis isolate CFT073 [19]. These isolates belong to the B2 clade of *E*. *coli*, the clade in which uropathogenic strains are most commonly found, but differ in their serotypes and their carriage of various mobile genetic elements, such as plasmids and pathogenicity islands that are known to harbor virulence genes [20–22]. We found that adaptive immunity was able to restrict chronic and same-strain recurrent cystitis caused by CFT073, but this restriction did not translate to cross-protection against UTI89 challenge infection. A better understanding of the complexities of the adaptive immune response to UTI could ultimately allow the development of new therapeutics, and inform our understanding of adaptive immunity in the context of chronic bacterial infections in general.

## Results

### Cystitis outcomes are bacterial strain-dependent

**Fig 2:**
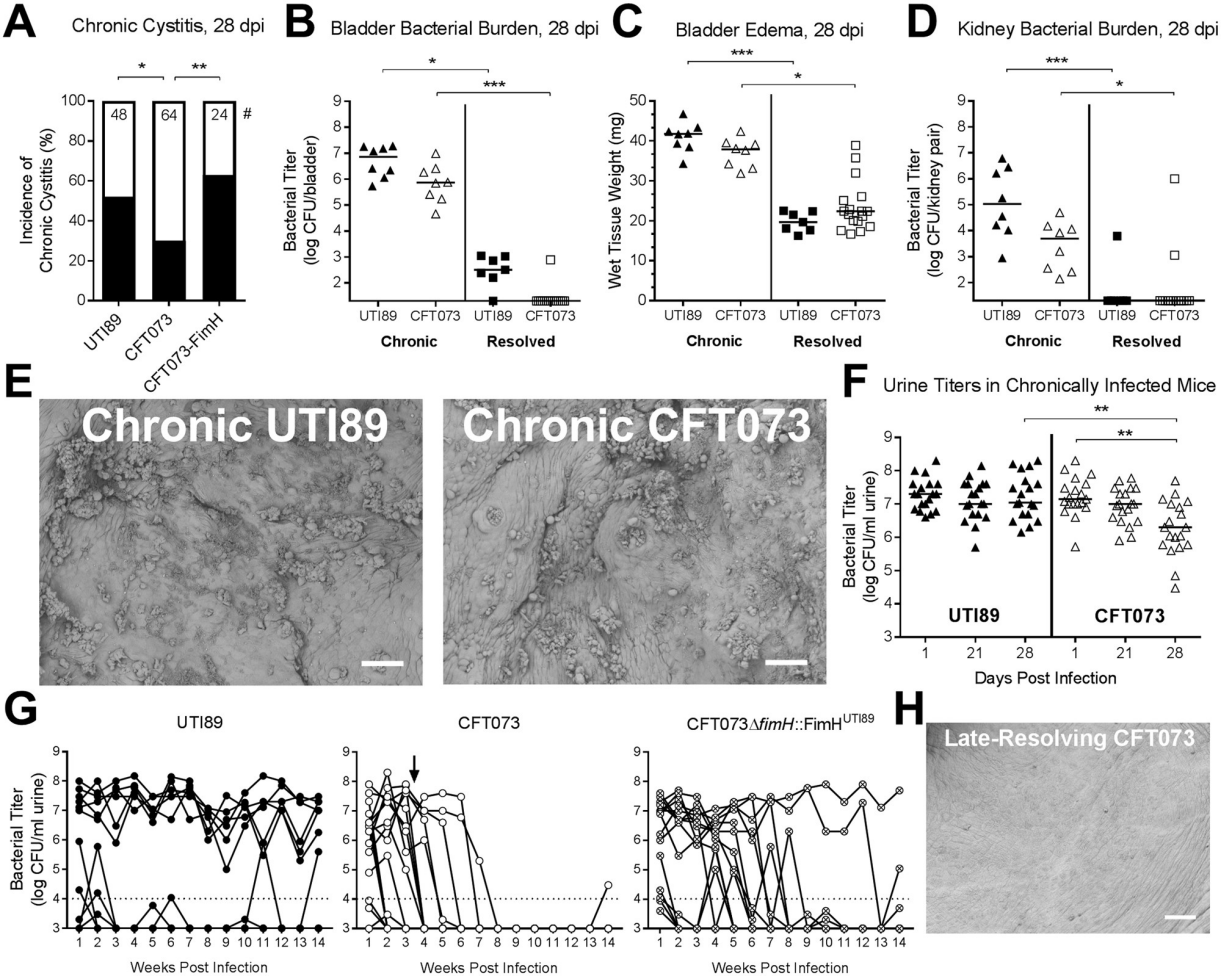
CFT073 infection is less robust than UTI89 at 28 dpi and clears over time. Juvenile Naive (7–8 week old) female C3H/HeN mice were infected with 10^8^ CFU UTI89, CFT073, or CFT073Δ*fimH*::FimH^UTI89^ (“CFT073-FimH”) and monitored for 28 days. (**A**) Percentage of mice (infected as indicated) that developed chronic cystitis, which was defined as persistent, high-titer bacteriuria (>10^4^ CFU/ ml urine), bladder bacterial burden >10^4^ CFU/bladder and bladder inflammation at time of sacrifice [14]. * *P* < 0.05, ** *P* < 0.01, Fisher’s exact test. (**B-D**) Bacterial burdens (**B**) and edema (**C**) and kidney burdens (**D**) at 28 dpi in mice with chronic cystitis caused by UTI89 or CFT073 (“Chronic”) compared to mice that had spontaneously resolved these infections (“Resolved”). **(E**) Scanning electron microscopy (SEM) of bladders harvested from mice with chronic UTI89 or chronic CFT073 cystitis at 28 dpi. Representative images from N = 1 experiment with n = 3 mice per group are shown; scale bars, 50 μm. (**F**) UTI89 and CFT073 urine titers over time in mice meeting the criteria for chronic cystitis (persistent, high-titer bacteriuria >10^4^ CFU/ ml urine). (**G**) Mice infected with UTI89 (n = 12), CFT073 (n = 18), or CFT073Δ*fimH*::FimH^UTI89^ (n = 15) were followed for 14 weeks. Dashed lines indicate our previously established cutoff for high-titer persistent bacteriuria, 10^4^ CFU/ml urine. Note that 4 out of 18 CFT073-infected mice resolved the infection between weeks 3 and 4 (indicated by the arrow); this “late-resolving” phenotype contributes to the lower incidence of CFT073 chronic cystitis at 4wpi, seen in **2A**. (**H**) SEM at 28 dpi of a mouse that resolved a CFT073 infection between days 21 and 28 (a so-called “late resolver”); scale bar, 50 μm. Data are combined from two to three independent experiments; data points represent actual values for each individual mouse, zeros are plotted at the limit of detection, and bars indicate median values. * *P* < 0.05, ** *P* < 0.01, *** *P* < 0.001, **** *P* < 0.0001, Kruskal-Wallis test with Dunn’s multiple test correction.

First we assessed differences in host outcomes 28 days after infection with 10^8^ CFU of either UTI89 or CFT073 (Fig 1C and 1D). We found that CFT073 caused chronic cystitis lasting four weeks, but at a significantly lower rate than UTI89 did (30% incidence for CFT073 vs. approximately 50% incidence with UTI89; Fig 2A), consistent with a previous study [21]. Whether infected with UTI89 or CFT073, mice with chronic cystitis at 28 dpi had significantly higher bladder bacterial burdens (Fig 2B) and bladder edema (Fig 2C) than mice that spontaneously resolved their infection, in accordance with our definition of chronic cystitis. Kidney titers were also higher in mice with chronic cystitis than those that resolved their infection (Fig 2D). In a blinded analysis of scanning electron micrographs of chronically infected bladders at 28 dpi, there were no obvious differences between chronic UTI89 and chronic CFT073 cystitis, with both infections causing epithelial disruption, immune cell infiltration, and abundant extracellular rod-shaped bacteria (Fig 2E). However, compared to chronic UTI89 cystitis, chronic CFT073 cystitis at 28 dpi trended toward reduced: bladder titers (Fig 2B), bladder edema (Fig 2C), and kidney titers (Fig 2D), suggesting that chronic CFT073 cystitis at this time point may be less robust than chronic UTI89 cystitis.

During chronic cystitis, CFT073 urine bacterial titers dropped significantly between 1 and 28 days post infection (dpi), though they remained above our predefined cutoff of 10^4^ CFU (Fig 2F). As well, in the course of this study we repeatedly observed that a subset of mice resolved their CFT073 infection between 21 and 28 days (i.e. persistent bacteriuria >10^4^ CFU until 21 dpi, but urine and/or bladder titers <10^4^ at 28 dpi), which we dubbed “late resolution.” Late resolution was not observed with UTI89 infection. Extending our studies past 28 days of infection revealed that every CFT073-infected mouse cleared bacteriuria within eight weeks (Fig 2G; note that four of 18 mice had “late resolution” between weeks three and four). In contrast, urine titers remained persistently high for 14 weeks post infection in the UTI89-inoculated mice that had developed chronic cystitis (Fig 2G). Analysis of a late-resolving CFT073-inoculated bladder by SEM revealed a calm surface at 28 dpi, with an intact epithelium having no immune cells or visible bacteria (Fig 2H).

A critical distinction between UTI89 and CFT073 is each strain’s allele of the mannose-binding *fimH* adhesin, which tips the type 1 pilus and mediates bladder colonization [23]. UTI89 has the FimH variant A62/V163, whereas CFT073 has the FimH variant S62/A163, which has a lower *in vitro* affinity for mannose and reduced virulence in the mouse bladder [21]. We found that a strain of CFT073 that expresses UTI89’s higher mannose affinity *fimH* allele, CFT073Δ*fimH*::FimH^UTI89^, had a significantly greater incidence of chronic cystitis at 28 dpi than wild-type CFT073 did (Fig 2A), in line with previous findings [21]. Strikingly, this strain was able to persist longer in the bladder than wild-type CFT073, but nevertheless all but one mouse eventually cleared the infection over the course of 14 weeks (Fig 2G). These observations indicate that while the high mannose affinity variant FimH enhances and prolongs the colonization of CFT073Δ*fimH*::FimH^UTI89^ in the mouse urinary tract relative to the wild type strain, other factors unique to CFT073 account for its propensity for being cleared from the host. As well, in the course of this work we became aware that the CFT073 strain used has a frameshift mutation in *rpoS* [24]. However, a CFT073 strain with a wildtype *rpoS* allele still had “late resolution” between days 21 and 28, and was cleared from all mice within eight weeks (S1 Fig), indicating that the *rpoS* frameshift mutation does not explain CFT073 clearance relative to UTI89.

### An acute checkpoint predicts chronic CFT073 infection, which leaves an imprint on the bladder

**Fig 3:**
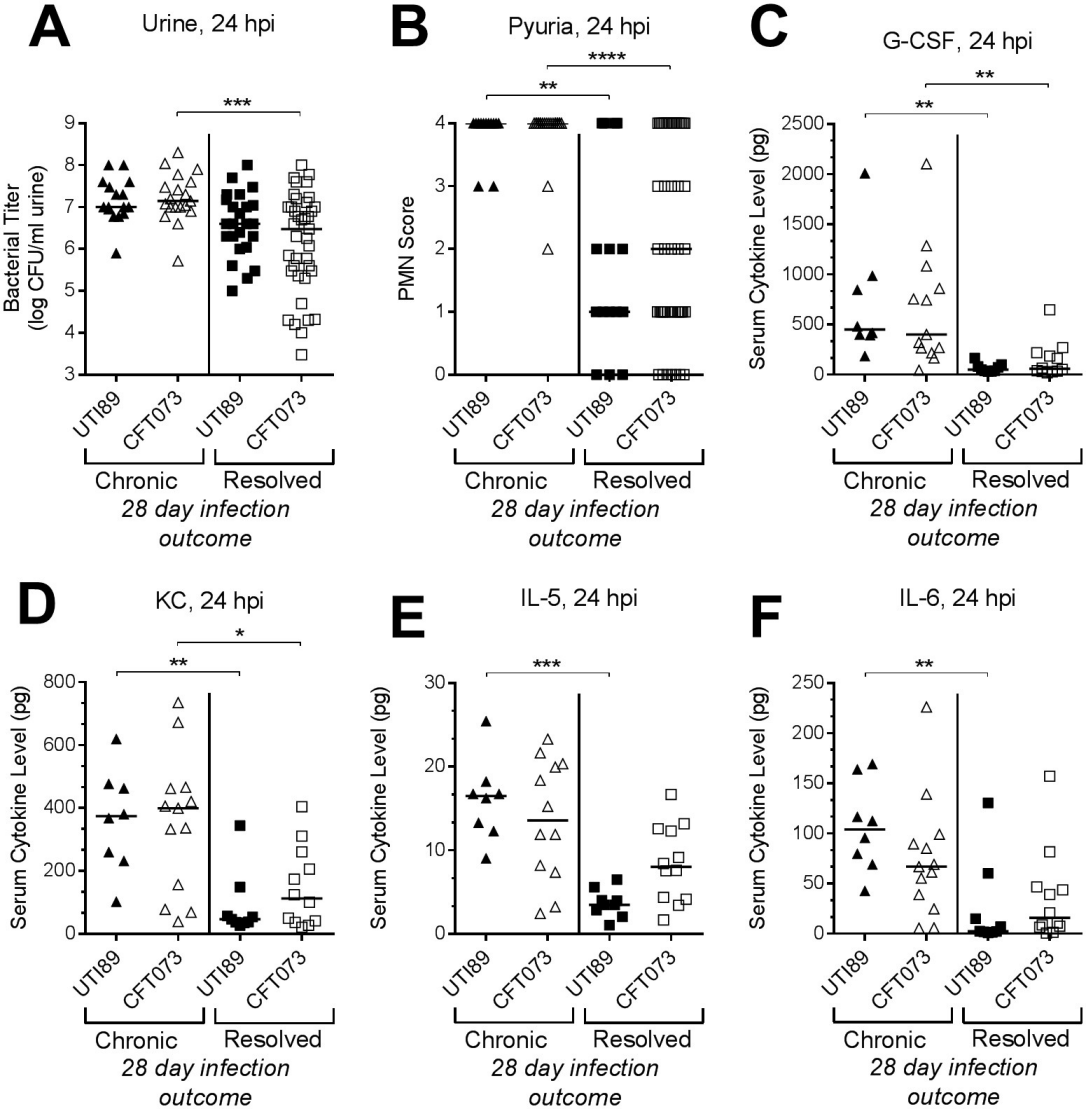
An acute host-pathogen checkpoint predicts the outcome of CFT073 bladder infection. At 24 hpi, urine and serum was collected from mice infected with 10^8^ CFU UTI89 or CFT073, and urine bacterial burden (**A**), pyuria (**B**) and serum cytokines (**C-F**) were assessed. Infection outcomes (chronic cystitis vs. resolution) were determined over the course of four weeks, and data are plotted according to the four week infection outcome. There were no statistically significant differences in the overall data (i.e. all UTI89-infected mice vs. all CFT073-infected mice) at 24 hpi. Data are combined from two to three independent experiments. Data points represent actual values for each individual mouse, zeros are plotted at the limit of detection, and bars indicate median values. * *P* < 0.05, ** *P* < 0.01, *** *P* < 0.001, **** *P* < 0.0001, Kruskal-Wallis test with Dunn’s multiple test correction. PMN, polymorphonuclear neutrophil.

We have previously shown that chronic UTI89 cystitis in C3H/HeN mice is predicted by an immune checkpoint at 24 hours post infection (Fig 1B), and that chronic bladder inflammation lasting four weeks results in a bladder imprint that persists after antibiotic therapy (Fig 1E). At 24 hpi, mice infected with CFT073 or UTI89 did not significantly differ in overall urine bacterial titers, pyuria, or pro-inflammatory serum cytokines. However, when mice were grouped by disease outcome, regardless of whether infected with UTI89 or CFT073 those that ultimately would develop chronic cystitis lasting four weeks had activation of the immune checkpoint, indicated by: higher urine bacterial burdens (Fig 3A), though here just a trend with UTI89; greater pyuria (Fig 3B); and increased serum levels of G-CSF and the IL-8 analogue KC, relative to the mice that would resolve their infection (Fig 3C and 3D). As well, there were trends toward higher serum IL-5 and IL-6 levels in mice that would develop chronic CFT073 cystitis (Fig 3E and 3F), but, unlike with UTI89-infected mice, the differences were not statistically significant. Thus, while we saw some minor differences between UPEC strains, checkpoint activation predicts the development of chronic cystitis lasting four weeks for both UTI89 and CFT073 in a similar manner.

**Fig 4:**
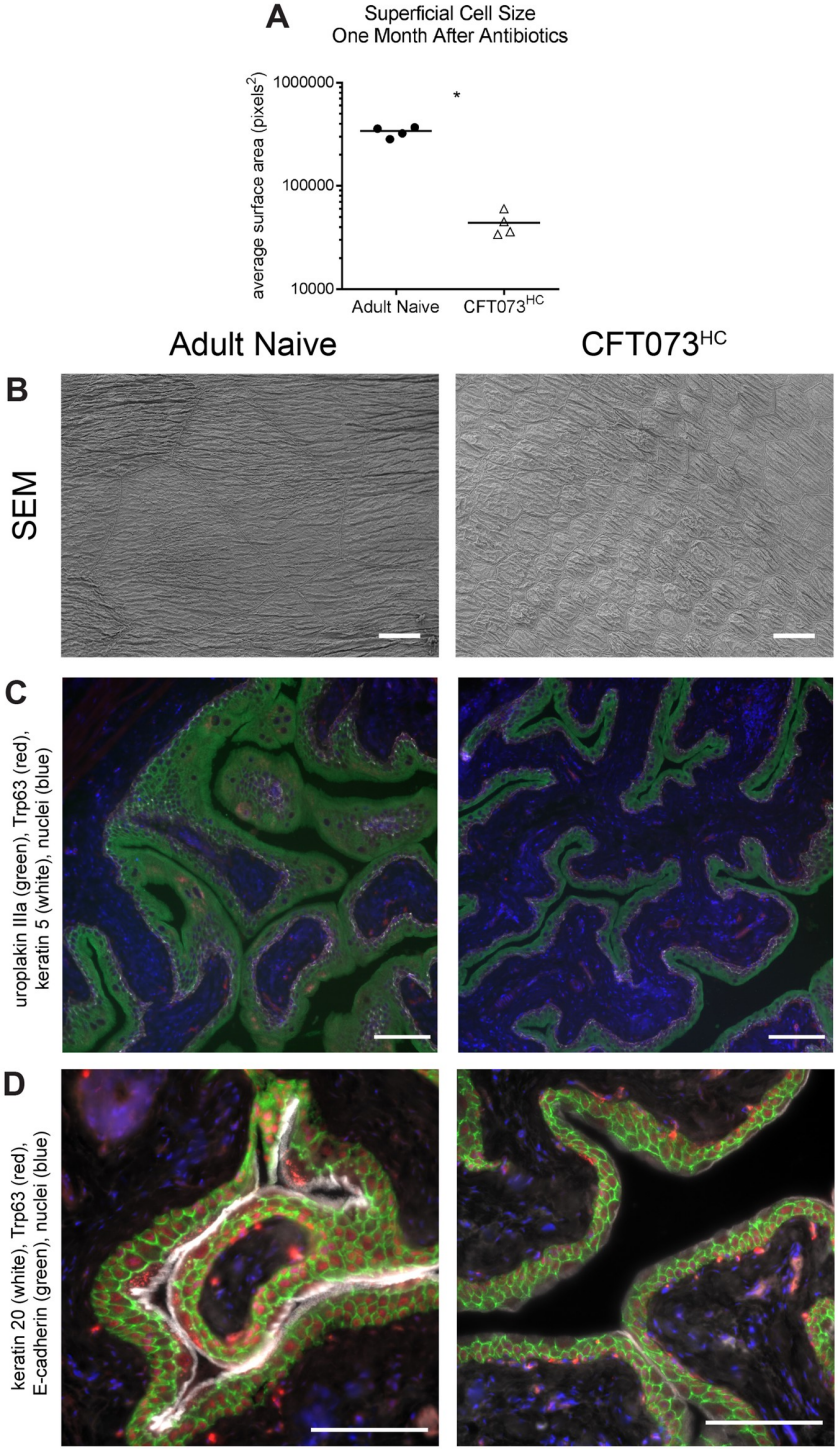
Chronic CFT073 cystitis causes bladder remodeling phenotypes that persist after antibiotic therapy. Mice were initially mock-infected (“Adult Naive”), or infected with 10^8^ CFU CFT073, and followed for four weeks, at which time ten days of antibiotics were initiated. Four weeks after the start of antibiotic therapy, convalescent bladders were harvested and assessed by microscopy. (**A and B**) Scanning electron microscopy (SEM) was used to assess bladder epithelial (urothelial) superficial cell size. (**A**) Superficial cells from N = 2 replicates with a total of n = 4 mice per group were measured in ImageJ and the average surface area (pixels^2^) was calculated. Data points represent the average of all measurements for a given mouse. * *P* < 0.05, Mann-Whitney U test. (**B**) Representative images from the analysis in (**A**). Scale bars, 25 μm. (**C and D**) Cell morphology and differentiation was assessed via immunofluorescence of paraffin-embedded bladder sections from N = 2 staining experiments with bladder sections from n = 3 Adult Naive mice and N = 5 CFT073^HC^ mice; representative images are shown. In (**C**) uroplakin IIIa is in green, Trp63 in red, keratin 5 in white and nuclei in blue. Scale bars, 50 μm. In (**D**), keratin 20 is shown in white, E-cadherin in green, Trp63 in red and nuclei in blue. Scale bars, 100 μm.

Scanning electron microscopy (SEM) analysis of bladders 28 days after antibiotic therapy showed that mice with a history of chronic CFT073 infection, referred to herein as CFT073^HC^ mice, had significantly smaller urothelial cells (average cell surface area approximately one eighth the size of Adult Naive cells) (Fig 4A and 4B), similar to the previously observed reduced size of urothelial cells from mice with a history of chronic UTI89 (UTI89^HC^) infection [15]. This change in bladder cell morphology is long-lasting in UTI89^HC^ mice: SEM analysis performed six months after antibiotics showed that UTI89^HC^ urothelial cells were still about one fourth the size of age-matched naive controls (S2A and S2B Fig).

Immunofluorescence microscopy of bladder sections revealed that while CFT073^HC^ mice expressed the terminal differentiation marker uroplakin IIIa (Fig 4C), expression of the terminal differentiation marker keratin 20 was weak and patchy, in line with previous observations for UTI89^HC^ mice [15] (Fig 4D). Weak and patchy keratin 20 staining was also found six months after initial infection in UTI89^HC^ mice (S2C Fig), suggesting that these infection-induced changes to the host may be permanent. Finally, histological examination of bladder sections from CFT073^HC^ mice 28 days after antibiotic therapy revealed the presence of lymphoid follicles (S3 Fig), which were previously found to develop between two and four weeks post infection in UTI89^HC^ mice [14]. Thus, chronic CFT073 infection results in a constellation of bladder remodeling phenotypes similar to those seen after chronic UTI89 infection, suggesting that bladder remodeling is a broad consequence of chronic *E*. *coli* infection.

### The ability of UPEC to cause recurrent UTI is bacterial strain-dependent

**Fig 5:**
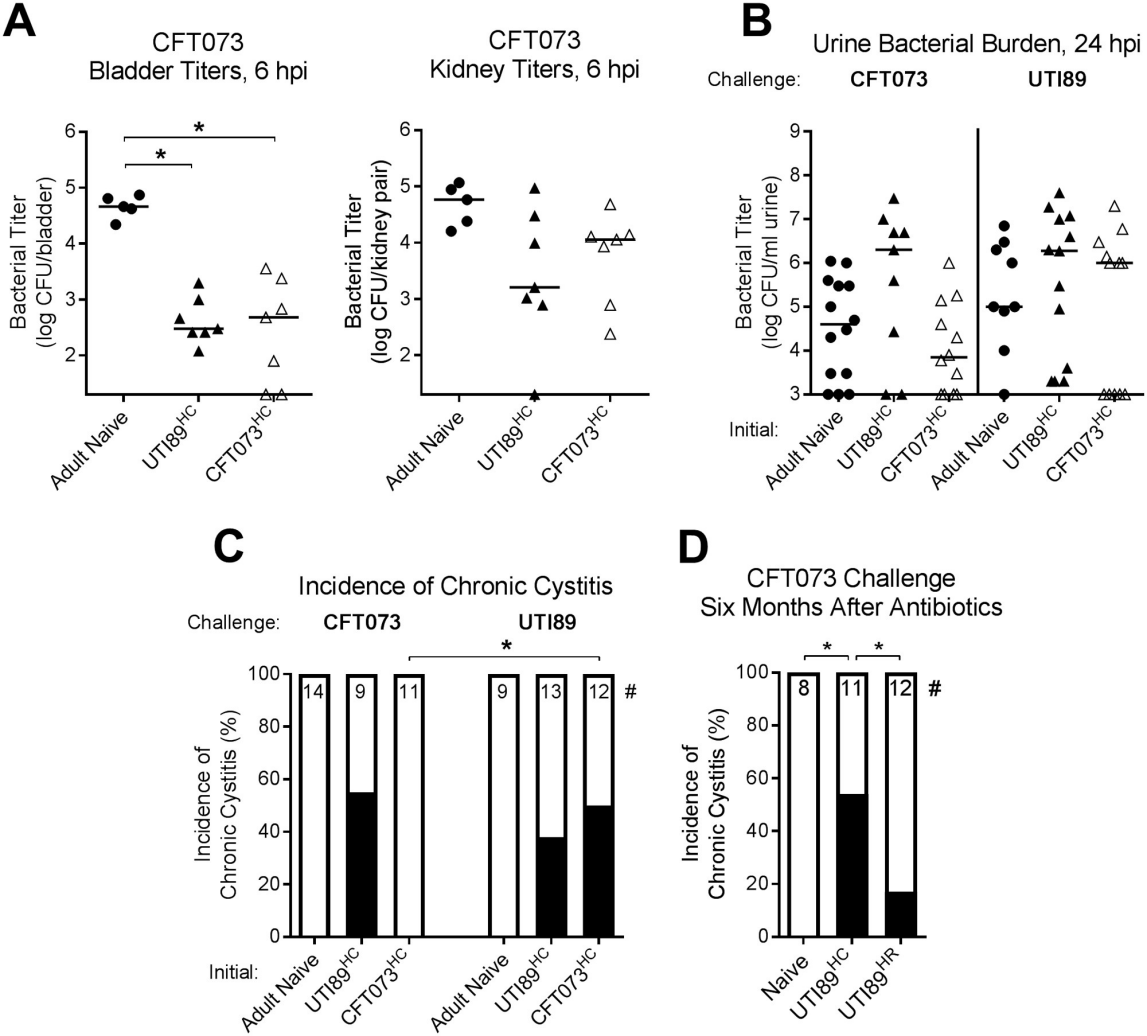
CFT073^HC^ mice are protected against CFT073 rUTI, but not against UTI89 rUTI. Mice were initially infected with 10^8^ CFU UTI89 or CFT073 and monitored over four weeks, at which time antibiotic therapy was initiated. Those mice that developed chronic cystitis during the initial infection were then challenged four weeks after antibiotics (**A-C**) or six months after antibiotics (**D**) with 10^7^ CFU UTI89 or CFT073 as indicated. Initially mock-infected mice were included as a control (“Adult Naive”). (**A**) Mice were sacrificed six hours after infection with CFT073 and bladder and kidney titers were assessed. * *P* < 0.05, Kruskal-Wallis test with Dunn’s multiple test correction. (**B and C**). Mice were challenged with UTI89 or CFT073 and followed over four weeks. (**B**) Urine titers at 24 hours post challenge, representing acute cystitis. (**C**) Incidence of chronic cystitis over the four week challenge. * *P* < 0.05, Fisher’s exact test. (**D**) Incidence of chronic cystitis over the four week challenge, six months after the initiation of antibiotics, compared to age-matched (“Naive”) mice and mice that had resolved their initial infection (“UTI89^HR^”). * *P* < 0.05, Fisher’s exact test. # denotes the number of mice per group. Data are from two to three independent experiments. Data points represent actual values for each individual mouse, zeros are plotted at the limit of detection, and bars indicate median values.

C3H/HeN mice become resistant to UPEC UTI with age [14,15,25], but UTI89^HC^ mice have enhanced susceptibility to UTI relative to age-matched (so-called “Adult”) Naive mice, a phenomenon we have termed “sensitization” to rUTI [14] (Fig 1G). To assess whether CFT073^HC^ mice were also sensitized to rUTI, mice with an initial chronic or mock infection were given antibiotics at four wpi and then challenged four weeks later with 10^7^ CFU of an isogenic strain with a different antibiotic resistance marker. Acute and chronic rUTI outcomes were then assessed. Previously, we found that UTI89^HC^ mice displayed a colonization resistance phenotype within the first 12 hours after challenge infection: UTI89 was less able to invade and replicate within remodeled bladder epithelial cells relative to naive ones, possibly due to their smaller cell size, resulting in significantly lower CFUs in UTI89^HC^ mice relative to age-matched Adult Naive mice [15]. We found that CFT073^HC^ mice also displayed a colonization resistance phenotype, with significantly reduced CFUs six hours after CFT073 infection in both UTI89^HC^ and CFT073^HC^ mice compared to age-matched Adult Naive mice (Fig 5A). Kidney titers were not statistically significantly different at this time point (Fig 5A). Despite early colonization resistance, UTI89 is nevertheless able to cause severe UTI at 24 hpi in about half of UTI89^HC^ mice, as indicated by high urine titers (median titer ~10^6^ CFU/ml) (Fig 5B) that precede the development of recurrent chronic cystitis over the 28 day challenge (Fig 5C). In contrast, CFT073 is not able to cause severe UTI in CFT073^HC^ mice, with median urine titer ~10^4^ CFU/ml urine at 24 hpi (Fig 5B) and zero incidence of chronic cystitis during the 28 day challenge (Fig 5C). Notably, CFT073^HC^ mice were only protected from same-strain challenge: UTI89 titers were high in the urine at 24 hpi (median titer ~10^6^ CFU/ml) (Fig 5B) and caused recurrent chronic cystitis in 50% of CFT073^HC^ mice (Fig 5C and S4 Fig). Likewise, UTI89^HC^ mice were susceptible to recurrent CFT073 cystitis (Fig 5B and 5C and S4 Fig), in line with previous observations [15], and remained susceptible to CFT073 for at least six months after antibiotic therapy (Fig 5D). Thus, whereas a history of UTI89 chronic cystitis predisposes to rUTI caused by either UTI89 or CFT073, a history of chronic CFT073 cystitis predisposes to rUTI caused by UTI89, but not caused by CFT073.

### T cell depletion abrogates protection from rUTI in a bacterial strain-dependent manner

The observations that chronic CFT073 infection resolves over time (Fig 2) and confers protection against CFT073 challenge (Fig 5) suggest that chronic CFT073 cystitis may elicit an adaptive immune response that clears the infection and protects against challenge. Thus we questioned whether components of the adaptive immune system played a role in this model. We assessed bacterial-specific IgG levels in the serum and found that both CFT073 and UTI89 elicited antibody responses, though anti-CFT073 IgG levels were somewhat higher than anti-UTI89 IgG levels (S5 Fig). We also tested whether T cell depletion would affect chronic and/or recurrent cystitis outcomes. For other chronic Gram-negative bacterial infections, depletion of both CD4^+^ and CD8^+^ T cells resulted in significantly worse infection phenotypes than single depletions did [26–29]. Hence, we tested the effects of both single subset depletions and combined depletions, compared to an equivalent dose of isotype (0.5 or 1 mg, respectively), during an initial UPEC infection. Depletion of lymphoid subsets and effects on myeloid subsets was determined four weeks after the initial infection, and at the time of the challenge infection, by flow cytometry of spleen single cell suspensions (S6 Fig).

**Fig 6:**
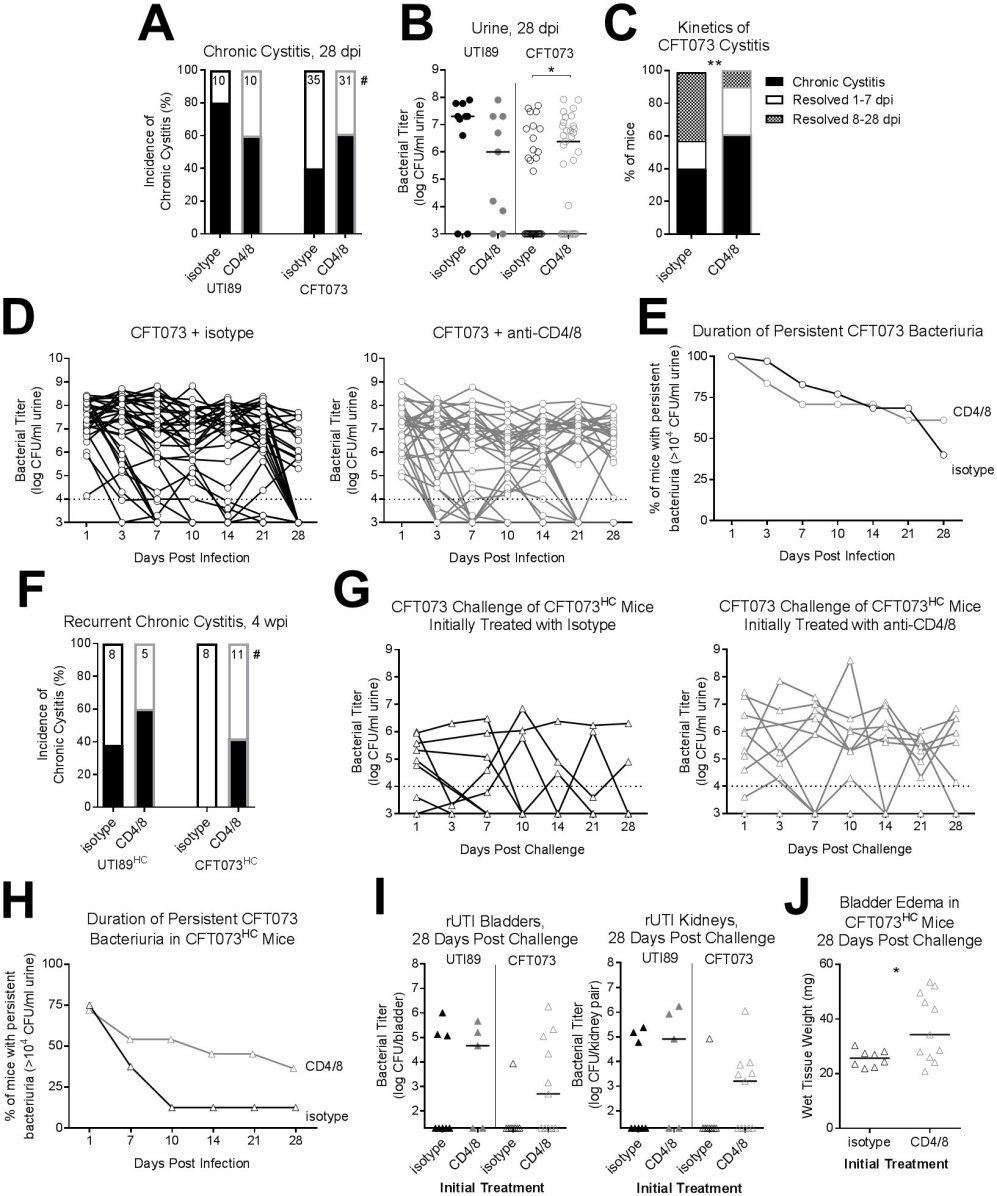
Depletion of CD4^+^ and CD8^+^ T cell subsets increases chronic and recurrent CFT073 cystitis. Mice infected with 10^8^ CFU UTI89 or CFT073 were given weekly doses of anti-CD4 and anti-CD8 antibodies, or isotype control, and followed for four weeks. (**A**) The incidence of chronic cystitis was determined at 28 dpi; # indicates the number of mice per group. (**B**) Urine bacterial burdens at 28 dpi. (**C**) CFT073-infected mice from (**A**) were binned according to the length of their infection. ** *P* < 0.01, Fisher’s exact test for resolving 8–28 dpi. (**D**) CFT073 urine titers over the course of infection from isotype-treated (left panel) and anti-CD4/8-treated (right panel) mice. Dashed lines indicate our previously established cutoff for high-titer persistent bacteriuria, 10^4^ CFU/ml urine. (**E**) Shown are the percentages of mice with persistent high-titer CFT073 bacteriuria (>10^4^ CFU/ml urine) over time. (**F-J**) At 28 dpi, antibiotic therapy was initiated. Those mice that developed chronic cystitis during the initial infection were then challenged four weeks after antibiotics with 10^7^ CFU of the same strain as their initial infection. Depletion antibodies were not administered during the challenge infection. (**F**) The incidence of recurrent chronic cystitis was determined at four weeks post challenge; # indicates the number of mice per group. (**G**) CFT073 urine titers over the course of the challenge infection in CFT073^HC^ mice, from mice initially isotype-treated (left panel) and initially anti-CD4/8-treated (right panel). Some samples were unavailable at three days post challenge. (**H**) Shown are the percentages of CFT073^HC^ mice with persistent high-titer recurrent CFT073 bacteriuria (>10^4^ CFU/ml urine) during the challenge infection. (**I and J**) Shown are bladder and kidney bacterial burdens (**I**) and bladder edema (**J**) at time of sacrifice. Data are combined from two to four independent experiments; data points represent actual values for each individual mouse, zeros are plotted at the limit of detection, and bars indicate median values. *, *P* < 0.05, Mann-Whitney U test. rUTI, recurrent urinary tract infection.

Depletion of CD4^+^ and CD8^+^ T cell subsets did not affect the incidence of chronic cystitis caused by UTI89 (Fig 6A). For CFT073 infection, the incidence of chronic cystitis at 28 dpi was higher in the single depletion groups (67% for CD4-depleted and 62% for CD8-depleted, compared to 46% in isotype-treated mice, S7A and S7B Fig), but the difference was not statistically significant, and similar proportions of mice cleared the infection after seven days (S7C Fig). Furthermore, mice depleted of single T cell subsets were still protected from same-strain rUTI (S7D and S7E Fig). In the combined CD4^+^ and CD8^+^ T cell depleted group, the rate of chronic CFT073 cystitis was 61%, vs. 40% in the isotype-treated group (Fig 6A), and CFT073 urine titers at 28 dpi were significantly elevated in the combined T cell-depleted mice relative to isotype-treated controls (Fig 6B). Notably, significantly fewer mice depleted of both CD4^+^ and CD8^+^ T cell subsets resolved the CFT073 infection after 7 dpi, relative to isotype-treated mice (Fig 6C). Accordingly, combined CD4^+^ and CD8^+^ T cell depletion also prevented the so-called “late resolver” phenotype (wherein some mice resolve their CFT073 infection between days 21 and 28 post infection; see Fig 2G). In the T cell-depleted group, n = 12 mice resolved the CFT073 infection: nine resolved within the first week and none between days 21 and 28. In contrast, of the n = 21 isotype-treated mice that resolved the CFT073 infection, ten resolved between days 21 and 28 (Fig 6D and 6E).

Based on the above results, we tested whether the abrogation of CD4^+^ and CD8^+^ T cell responses during the initial CFT073 infection would render mice susceptible to recurrent CFT073 cystitis. Four weeks after the initial infection and concomitant T cell depletion, mice were given antibiotics and then challenged four weeks later with the same strain as their initial infection, without additional T cell depletions. UTI89^HC^ mice were susceptible to developing recurrent UTI89 cystitis, whether they were initially treated with anti-CD4/8 or isotype (Fig 6F). In contrast, T cell depletion had a striking effect on CFT073 challenge of CFT073^HC^ mice. No mice (out of n = 8) in the isotype-treated group developed recurrent chronic CFT073 cystitis, consistent with our observations in untreated mice (see Fig 5C); one mouse had persistent bacteriuria but had a bladder titer <10^4^ CFU. However, four of 11 mice that received anti-CD4/8 during their initial chronic infection developed recurrent chronic CFT073 cystitis lasting four weeks (Fig 6F–6H) and one mouse was a “late resolver,” similar to what is seen during CFT073 infection of juvenile naive mice (see Figs 2G and 6D). Bladder and kidney bacterial burdens did not differ between isotype vs. anti-CD4/8 treatments in UTI89^HC^ mice after UTI89 challenge, but in CFT073^HC^ mice challenged with CFT073, titers at sacrifice were higher in those that received anti-CD4/8 than those that received isotype control (Fig 6I). As well, bladder edema four weeks post challenge with CFT073 was significantly greater in CFT073^HC^ mice that received anti-CD4/8 than in those that received isotype control (Fig 6J). In sum, these experiments show that depletion of both CD4^+^ and CD8^+^ T cell subsets during an initial CFT073 infection renders C3H/HeN mice susceptible to chronic and same-strain recurrent cystitis, whereas T cell depletion during a UTI89 infection does not impact chronic or same-strain recurrent cystitis.

## Discussion

Some women who suffer from an acute UTI will become afflicted by highly recurrent UTI, but most women do not experience this fate. It may be that adaptive immunity protects at least some women from rUTI, but relatively little is known about adaptive immune responses to cystitis. We showed that the clinical urosepsis isolate CFT073 can cause severe acute and chronic UTI in C3H/HeN mice. In line with our previous findings for the clinical UTI isolates UTI89 and J96 [14], chronic CFT073 cystitis developed after the triggering of an acute host-pathogen checkpoint, and chronic infection lasting four weeks resulted in bladder mucosal remodeling. Thus, we suggest that checkpoint activation is broadly predictive of chronic UPEC UTI, and that bladder mucosal remodeling is a general consequence of chronic UPEC infection and/or infection-associated inflammation. Unlike UTI89, however, CFT073 cystitis did not last indefinitely; all mice cleared CFT073 infection within eight weeks, and after an initial chronic infection lasting four weeks, mice were protected from CFT073 challenge, but susceptible to UTI89 challenge, demonstrating strain-specific protection. We found that the combined depletion of CD4^+^ and CD8^+^ T cell subsets during the initial CFT073 infection increased the incidence of chronic cystitis and rendered mice susceptible to recurrent chronic CFT073 cystitis. T cell depletion did not significantly impact chronic or recurrent cystitis caused by UTI89, in line with a previous study of recurrent UTI in C57BL/6 mice [13]. Thus, this study provides evidence for a role of T cells and adaptive immunity in restricting chronic and same-strain recurrent UTI caused by some, but not all, UPEC strains.

Placebo-controlled studies have demonstrated two general outcomes of uncomplicated cystitis: while many women can resolve their infections without the aid of antibiotics, others remain bacteriuric (with or without symptoms) for weeks [30], suggesting an ineffective or absent adaptive response. As well, we note that spontaneous resolution of infection in placebo-treated women has been reported to occur both early in infection (within seven days) and later on (between four to seven weeks) [6]. Much is known about innate immune responses to UTI [10,11], but fewer studies have focused on the role of adaptive immunity. In our study we recapitulated the two general cystitis outcomes seen in women by using inbred C3H/HeN mice, which can spontaneously resolve bladder infections or develop chronic cystitis lasting two or more weeks. A role for adaptive immunity was elucidated via depletion of T cells, which skewed the balance of infection outcomes in a bacterial strain-specific manner. In T cell-depleted mice, the incidence of chronic CFT073 cystitis was increased due to the elimination of the “late resolvers” (mice that resolved a chronic infection after 21 days), while chronic UTI89 cystitis was not affected because late resolution does not occur after infection with this strain. Our finding of strain-specific adaptive immunity echoes the recent discovery that different clinical isolates of the Gram-positive pathogens *Staphylococcus aureus* and *Streptococcus pyogenes* vary substantially in their ability to elicit adaptive immune responses *in vitro* depending on the content of their accessory (non-core) genome [31].

It has previously been shown that adaptive responses can protect against same-strain rUTI in C57BL/6 mice or mutants on the C57BL/6 background [12,13,32]. The work presented herein elucidates the complexity of this response by showing bacterial strain-dependent variability. Studying multiple UPEC strains is critical because ongoing genome sequencing has revealed remarkable genetic diversity in *E*. *coli*, as the pan-genome remains “open” and continues to expand beyond more than 16,000 genes, resulting in the extremely variable accessory genome, typically comprised of secretory systems, metabolic pathways, and virulence gene networks [33]. A large-scale genomic study of 43 *E*. *coli* isolates from 14 women suffering from recurrent UTI [34] revealed that the isolates were phylogenetically diverse, as they represented five major *E*. *coli* clades and only about 60% of each strain’s genome was found to be shared among all of the strains. Upon infection in mice, significant heterogeneity in bladder titers was observed with these genetically diverse strains. Notably, some strains from clades that are not considered traditionally uropathogenic (such as B1) could robustly colonize the mouse bladder, while conversely, some strains from the B2 clade (typically considered uropathogenic) were poor colonizers of mice. Further, no single genetic signature predicted a strain’s ability to colonize the mouse bladder. This study indicated that a complex interplay between UPEC genetics and host susceptibility was necessary for UTI to develop, and that there may be multiple pathogenic pathways in UPEC that can lead to infection [34]. Based on our work here, we argue that depending on the host and the infecting strain, adaptive immunity may or may not be capable of restricting chronic and same-strain recurrent UTI. Further, even if an adaptive response restricts same-strain rUTI, it may not broadly protect against challenge with diverse UPEC strains.

The present study adds further proof of the complexity of UTI in the form of host adaptive immune responses that vary with the infecting strain. By comparing and contrasting different clinical UPEC isolates, we begin to recapitulate the great genetic and phenotypic diversity of uropathogens–and thus, bacterial antigens–to which women are exposed. During chronic cystitis the host is likely to have significant exposure to the highly immunogenic UPEC outer membrane components that comprise the classical bacterial serotype: the O antigen polysaccharide of LPS; the K capsule; and the H antigen, flagellin. The components of the *E*. *coli* serotype, defined by their ability to generate an immune response in a diagnostic setting, are numerous: as of 2003, 170 O antigens, 103 K antigens, and 56 H antigens had been identified [35]. Host immune responses acting against certain serotype components may present a selective pressure that drives the high diversity in serotype components. Although UTI89 and CFT073 are genetically relatively similar and both belong to the B2 clade [34], they have different serotypes: UTI89 is O18:K1:H7 while CFT073 is O6:K2:H1 [20]. Interestingly, a previous study found that experimental UTI with an *E*. *coli* O6:K13:H1 strain elicited a T cell response in rats [36]. One or more components of the UTI89 serotype may be able to subvert adaptive immunity to facilitate long-term persistence and same-strain recurrence.

Depletion of both CD4^+^ and CD8^+^ T cell subsets during the initial infection was necessary to prevent “late resolution” of chronic CFT073 cystitis and render the host susceptible to recurrent chronic CFT073 cystitis, suggesting that one subset may compensate for the loss of the other. A similar paradigm was observed for infection with the Gram-negative intracellular pathogen *Francisella tularensis*, wherein studies using T cell depletions or T cell knockout mice have demonstrated that while mice that have lost either CD4^+^ or CD8^+^ T cells are able to clear an initial or challenge infection, the loss of both CD4^+^ and CD8^+^ T cell subsets impairs bacterial clearance and protective memory responses [29,37,38]. CD4^+^ or CD8^+^ T cells could control *F*. *tularensis* growth in macrophages *in vitro* in part through the production of IFN-γ [39], and *in vivo* both subsets produced IFN-γ [38]; interestingly, IFN-γ knockout mice on the C57BL/6 background were previously shown to be more susceptible to UTI than immunocompetent mice [40]. In our study of chronic and recurrent UTI, the mechanism(s) of T cell-mediated protection are as yet unknown. CD4^+^ T cells could play a variety of protective roles, including: i) directly stimulating neutrophil recruitment via IL-17 production; ii) activating macrophages and CD8^+^ T cells via IFN-γ; iii) supporting B cell affinity maturation and isotype switching; and/or iv) themselves becoming memory T cells [41]. CD8^+^ T cells could be directly cytotoxic against infected urothelial cells, and/or could be responding to cross-presentation of antigens by dendritic cells or other cell types; previously, macrophages were found to sequester UTI89 from dendritic cells, limiting adaptive responses to same-strain recurrent UTI in C57BL/6 mice [13]. We found that splenic monocytes and neutrophils, but not macrophages, were elevated in T cell-depleted mice at 28 dpi, which might suggest an expanded role for monocytes and neutrophils in host response in the absence of T cells. Further studies are needed to determine the mechanisms of T cell-mediated protection against specific UPEC strains, as well as to assess the role of B cells and antibodies in this UTI model. A previous study in C57BL/6 mice found that concurrent kidney and bladder infections led to a strong pathogen-specific serum IgG response, compared to bladder-restricted infections [42]. Here we observed that pathogen-specific serum IgG levels were higher in CFT073^HC^ mice than UTI89^HC^ mice. These data suggest that CFT073 is better able to induce an antibody response, though the exact reason for this difference is unclear at this point. Finally, γδ T cells were previously shown to play a role in protection against UTI in C57BL/6 mice [43]; our depletion antibodies are not expected to target γδ T cells, so their potential role in this model of chronic and recurrent UTI remains unexplored.

Although UTI are among the most common bacterial infections, most cases are uncomplicated and readily treated by antibiotics. However, our ability to treat these infections is threatened by the global rise of multidrug-resistant “superbugs”; indeed, recently the first United States case of *E*. *coli* resistant to the last line drug colistin was reported in a UTI patient [44]. It has become increasingly clear that rUTI result from complex interactions among host risk factors (both intrinsic, like genetic polymorphisms affecting UTI susceptibility, and extrinsic, i.e. behavioral risk factors such as sexual activity) [45] and genetically diverse uropathogens possessing an enormous variety of bacterial virulence factors [34]. Our study emphasizes the additional complexity of host immune responses to specific bacterial strains. A major question going forward will be what bacterial factor(s) govern the development or suppression of adaptive immunity in susceptible hosts. Many experimental UTI vaccines–including attenuated or inactivated whole bacteria as well as specific bacterial components–have elicited protection against UTI in animal models [46]. However, translating these preclinical studies to a successful and widely used clinical UTI vaccine has proven difficult. Our study demonstrates that the development of a broad spectrum vaccine will need to take into account the genetically diverse uropathogens that women encounter in the community, and emphasizes the importance of incorporating multiple bacterial strains and the effects of previous exposures in animal models of host-pathogen interactions and outcomes.

## Materials and methods

### Ethics statement

All mouse experiments were conducted according to the National Institutes of Health guidelines for the housing and care of laboratory animals. All experiments were performed in accordance with institutional regulations after review and approval by the Institutional Animal Care and Use Committee (IACUC) at Washington University School of Medicine in St. Louis, MO (animal protocol number 20150226).

### Bacterial strains

The uropathogenic *E*. *coli* isolates used in this study were the human cystitis isolate UTI89 [18] and the antibiotic-resistant derivatives UTI89 attHK022::COMGFP (kanamycin-resistant) and UTI89 attl::PSSH10-1 (spectinomycin-resistant) [47]; the human urosepsis isolate CFT073, wild-type and Δ*rpoS* [19], and the antibiotic-resistant derivatives CFT073Δ*rpoS* HK::Cm (chloramphenicol-resistant), CFT073Δ*rpoS* HK::Kan (kanamycin-resistant), and CFT073Δ*rpoS* Δ*fimH*::FimH^UTI89^ (kanamycin-resistant) [21]. Strains were cultured statically in lysogeny broth (LB) at 37°C for two to three overnight passages to induce type 1 pilus expression. In experiments where mice had an initial infection and subsequent challenge infection, the challenge strain was marked with different antibiotic resistance from the initial strain.

### Mouse infections

C3H/HeN mice were obtained from Envigo Research Model Services, Inc. (formerly Harlan). All mice were female and 7–8 weeks old (“Juvenile”) at the time of the first infection (or mock-infection in the case of the Adult Naive group). Bacterial inocula were prepared as previously described [14] and 10^7^ or 10^8^ CFU of bacteria were inoculated into the bladders of C3H/HeN mice by transurethral catheterization under isoflurane anesthesia as previously described [48]. For infections in Juvenile Naive mice, 10^8^ CFU was always used, since C3H/HeN mice develop chronic cystitis in an infectious dose-dependent manner. For challenge infections, 10^7^ CFU (of a differently antibiotic-marked strain than the initial infection) was used, to facilitate comparisons with prior studies [14,15,17]. To monitor infection outcomes, urine, bladder and kidney bacterial burdens were determined as previously described [14]. Mice were humanely euthanized by isoflurane inhalation followed by cervical dislocation. Chronic cystitis was defined as persistent high-titer bacteriuria (>10^4^ CFU/ml urine) at every urine collection time point (1, 3, 7, 10, 14, 21, and 28 days post infection, and weekly thereafter if experiments continued). A cutoff of 10^4^ CFU/ml persistent bacteriuria is highly specific and sensitive for the detection of chronic cystitis [14]. In addition, chronic cystitis was also indicated by a high bladder bacterial burden (>10^4^ CFU/bladder) and a visibly enlarged, inflamed bladder (indicating inflammation and edema) at time of sacrifice (≥28dpi). Resolution of cystitis was defined as urine bacterial titer dropping below 10^4^ CFU/ml urine during at least one time point, and/or bladder bacterial burden <10^4^ CFU at time of sacrifice. For long-term “challenge” experiments, beginning four weeks after the initial infection, all mice (including mock-infected controls, and regardless of infection outcome) were treated with trimethoprim and sulfamethoxazole in the drinking water (54 and 270 μg/ml water, respectively) for ten days. Urine samples were collected weekly after the initiation of antibiotics to confirm sterile urines and any mice with treatment failure were excluded from subsequent analysis. Four weeks or six months after the initiation of antibiotics to clear initial infection, mice were challenged with 10^7^ CFU of bacteria. The same criteria were used to define chronic cystitis during the challenge infection as in the initial infection (persistent bacteriuria >10^4^ CFU/ml urine; bladder burden >10^4^ CFU; enlarged bladder), with the exception that urine titers could be <10^4^ CFU/ml on the first day post challenge, as we have found that checkpoint activation in mice with a history of chronic cystitis may take longer (up to 72 hours) than in Juvenile Naive mice (24 hours).

### Histopathology and immunofluorescence

Bladders were aseptically harvested and fixed overnight in methacarn (60% methanol, 30% chloroform, 10% glacial acetic acid), bisected to give two halves per bladder, paraffin-embedded and sectioned. For histopathological examination, slides were stained with hematoxylin and eosin and imaged with a Zeiss Axio Scan Z.1 brightfield slide scanner. Immunofluorescence experiments were conducted as previously described [15]. Primary antibodies used were uroplakin IIIa (mouse monoclonal, 10R-U103a, Fitzgerald), Trp63 (rabbit polyclonal, GTX102425, GeneTex), E-cadherin (goat polyclonal IgG, AF748, R&D Systems), cytokeratin 5 (chicken polyclonal, 905901, BioLegend) and cytokeratin 20 (mouse monoclonal, M7019, DAKO). Samples were mounted in ProLong Gold Antifade Mountant with DAPI (ThermoFisher Scientific) and fluorescence was visualized on a ZEISS Axioskop Observer.Z1 microscope.

### Scanning electron microscopy

Two microscopy sample preparation protocols were used during the course of this work. For urothelial cell size determination six months after antibiotics, bladders were aseptically harvested, bisected, splayed in dissecting trays, and fixed in EM fixative as previously described [15]. The fixative comprised 2% paraformaldehyde and 2% glutaraldehyde in 0.1M sodium phosphate buffer, pH 7.4. Samples were prepared by critical point drying. Briefly, samples were post-fixed in 1% osmium tetroxide, dehydrated in increasing concentrations of ethanol, then dehydrated at 31.1°C and 1072 PSI for 16 minutes in a critical point dryer. Samples were mounted on carbon tape-coated stubs and sputter-coated with gold/palladium under argon. After these experiments, subsequent SEM samples were processed with a different protocol, which we verified gave the same results as prior studies. Thus, for all other experiments, the EM fix comprised 2% paraformaldehyde, 2.5% glutaraldehyde in 0.15M sodium cacodylate buffer with 2 mM CaCl_2_ at pH 7.4, and after post-fixing in 1% osmium tetroxide and critical point drying, samples were sputter-coated with iridium. For urothelial cell size determination after UTI89 vs. CFT073 infection, bladders were aseptically harvested, bisected, splayed in dissecting trays, and fixed in EM fixative before further processing. For the bladders obtained during chronic UTI89 or CFT073 cystitis at 28 dpi, the “balloon method” was used, wherein bladders were instilled with fixative prior to harvest, as previously described [49]. All bladders were imaged on a Zeiss Crossbeam 540 FIB-SEM or a Zeiss MERLIN FE-SEM. For urothelial cell size determinations, ImageJ 1.47v (National Institutes of Health, USA) was used to calculate epithelial cell surface area in five 500x magnification fields per bladder half (ten per mouse).

### Assessment of acute inflammation and checkpoint activation

Bacteria were enumerated in urine or bladder samples by serial dilution as previously described [14]. Urine sedimentation to determine neutrophil influx was performed as previously described [14]. Briefly, 80 μl of a 1:10 dilution of urine was centrifuged onto poly-L-lysine-coated glass slides and stained with a Hema 3 kit (Fisher Scientific). Slides were examined by light microscopy and the average number of polymorphonuclear leukocytes (PMN) per high-powered field (hpf; 400x magnification) was calculated from counting five fields. A semi-quantitative scoring system was used: 0, less than 1 PMN/hpf; 1, 1–5 PMN/hpf; 2, 6–10 PMN/hpf; 3, 11–20 PMN/ hpf; and 4, >20 PMN/hpf. Bladder edema was assessed by pre-weighing empty bladder homogenization tubes, weighing the bladder-containing tubes, and calculating the difference. Serum cytokines were assessed using the Luminex-based multiplex cytometric bead array platform (Bioplex, Bio-Rad, Hercules, CA) using beads individually purchased for IL-5, IL-6, KC, and G-CSF. The assay was performed according to the manufacturer’s instructions, except using 10-fold less standard and half the amount of coupled beads and detection antibodies indicated in the protocol, as described in [50]. Individual samples were run in duplicate and the mean value was used for plotting and statistical analysis.

### Serum ELISAs

Blood was obtained from convalescent mice (four weeks after the initiation of antibiotics) via submandibular puncture using a 5 mm lancet (Goldenrod) and collected in a BD Microtainer serum separator tube (BD Biosciences). UTI89 and CFT073 were grown as described above and 1 ml of OD_600_ = 1 bacteria was lysed by boiling at 100°C for 5 minutes. Lysed bacteria were diluted tenfold in PBS and applied to high-affinity Immulon 2 HB 96 well plates (Thermo Fisher) overnight at 4°C. Plates were washed with PBS containing 0.05% Tween 20 and a dilution series of serum was applied for 90 minutes at room temperature. After washing, a 1:10,000 dilution of HRP-conjugated goat anti-mouse IgG (Invitrogen) was applied for 90 minutes at room temperature. After a final wash step, the TMB substrate reagent set (BD Biosciences) was used to develop the reaction. Absorbance was measured on a VersaMax microplate reader (Molecular Devices).

### T cell depletions

Depletion antibodies were obtained from BioXCell: α-mouse CD4 (GK1.5, BP0003-1), α-mouse CD8a (2.43, BP0061), and rat IgG2b isotype control (LTF-2, BP0090). Mice were given intraperitoneal injections one day prior to infection and at days 7, 14, and 21 post infection. For depletions of both CD4^+^ and CD8^+^ T cell subsets, mice were treated with 500 μg α-CD4 and 500 μg α-CD8, or 1 mg isotype control, in a 500 μl volume of pH 7.0 dilution buffer (BioXCell). For single subset depletions, mice received 500 μg α-CD4 or 500 μg α-CD8, or 500 μg isotype control, in a 250 μl volume of pH 7.0 dilution buffer (BioXCell). T cell depletions were performed only during the initial infection, not the challenge infection.

### Flow cytometry

Spleens were dissected and placed in RPMI (Gibco) on ice until spleens were removed from all animals. Each sample was dissociated by placing the spleen between two frosted glass slides and rubbing the slides together. The spleens were placed in ACK buffer (Gibco) for 5 minutes at room temperature to lyse red blood cells. Ten mls of RPMI was added to each tube and tubes were inverted to mix, then centrifuged at 1300 rpm at 4°C for 5 minutes. The supernatant was discarded and the pellet was resuspended in 3 mls RPMI and pushed through a 70 μm cell strainer (Fisher). The cells were washed twice in 1X dPBS and centrifuged at 500 X g. The pelleted cells were resuspended in Fc block (BD Biosciences) and incubated in 1 μl of live/dead blue fluorescent reactive dye (Thermo Fisher/Invitrogen, L34962) per 10^6^ cells for 20 minutes at 4°C, then washed in 1X dPBS. Cells were stained with surface markers, all from Biolegend, in two separate panels: Panel 1: CD45 (30-F11), CD8a (53–6.7), CD3 (17A2), CD19 (GD5), and CD4 (GK1.5); Panel 2: CD45 (30-F11), CD11b (M1/70), Ly6G (1A8), F4/80 (BM8), and Ly6C (HK1.4). Sample data was acquired on the LSR II flow cytometer (BD Biosciences) recording 10,000 events. The data was analyzed using FlowJo software (version 10.0). The relative proportion of cellular infiltrates was expressed as a percentage of live cells.

### Statistical analysis

Our experience with the C3H/HeN chronic and recurrent cystitis model shows that when assessing bimodal responses, five mice per replicate (two replicates minimum) is the minimum number necessary to overcome any biological and/or technical variability and provide reliable and interpretable results within an experiment; a statistical analysis was not used to determine the number of mice per experiment. Investigators were not blinded to the infection history of the animals, except for Fig 2E. Statistics were performed in GraphPad Prism v7.01. A two-tailed Fisher’s exact test was used to test for significant differences in the incidence of chronic cystitis and kinetics of infection resolution. For bacterial titers, pyuria, cytokine expression levels, and surface area differences in superficial cells, comparisons of three or more groups were performed with the Kruskal-Wallis test followed by Dunn’s multiple test correction. Pairwise comparisons were performed with a two-tailed Mann-Whitney U test. *P* < 0.05 was considered statistically significant.

## Supporting information

S1 Fig The “late resolution” phenotype of CFT073 is not due to its *rpoS* allele. (**A**) Mice were infected with a strain of CFT073 with a wild-type *rpoS* allele, verified by sequencing using the primers in [24], and followed over four weeks. Data are from two experiments; data points represent actual values for each individual mouse and zeros are plotted at the limit of detection. (**B**) A subset of mice (not depicted in panel **A**) that were still chronically infected at four weeks were followed for an additional five weeks, during which time all mice resolved the infection.
(TIF)

S2 Fig The “molecular imprint” of chronic UTI89 cystitis lasts for at least six months after sterilizing antibiotic therapy. Mice were initially mock-infected (“Age-Matched Naive”), or infected with UTI89. Antibiotics were initiated at four wpi, and mice were allowed to convalesce for six months. (**A and B**) Scanning electron microscopy (SEM) was used to assess urothelial superficial cell size in the Naive mice vs. mice that initially had a chronic UTI89 infection (UTI89^HC^) or initially resolved their UTI89 infection (UTI89^HR^). (**A**) Representative images from N = 2 replicates with a total of n = 3–4 mice per group are shown. Scale bars, 25 μm. (**B**) Superficial cells from N = 2 replicates with a total of n = 3–4 mice per group were measured in ImageJ and the average surface area (pixels^2^) was calculated. Data points represent the average of all measurements for a given mouse. * *P* < 0.05, Kruskal-Wallis test with Dunn’s multiple test correction. (**C**) Immunofluorescence microscopy was performed on paraffin-embedded bladder sections from N = 2 staining experiments with bladder sections from n = 3 Adult Naive mice, N = 5 UTI89^HC^ mice, and N = 4 UTI89^HR^ mice; representative images are shown. Keratin 20 is shown in white, E-cadherin in green, Trp63 in red and nuclei in blue. Scale bars, 50 μm.
(TIF)

S3 Fig Chronic CFT073 infection results in lymphoid follicles. We previously observed that chronic UTI89 infection results in lymphoid follicles that persist after antibiotic therapy [14]. Here bladders were harvested from CFT073^HC^ mice four weeks after antibiotic therapy. Histological examination of hematoxylin & eosin-stained sections shows evidence of lymphoid follicles in the lamina propria of CFT073^HC^ mice (top; example circled in orange). Lymphoid follicles were not observed in mice that spontaneously resolved a CFT073 infection (bottom). Representative images from N = 2 replicates with a total of n = 4 mice are shown.
(TIF)

S4 Fig CFT073^HC^ mice are protected from same-strain rUTI but susceptible to UTI89 rUTI. Shown are the urine titers during challenge infection for mice in Fig 5B and 5C. Mice were initially infected with 10^8^ CFU UTI89 or CFT073, or mock-infected, and monitored over four weeks, at which time antibiotic therapy was initiated. Four weeks after antibiotics, mice were challenged with 10^7^ CFU CFT073 or UTI89. We note the instance of two “late resolvers” in the initially mock-infected (Adult Naive) mice challenged with CFT073 (see Fig 2G). Data are combined from two to three independent experiments; data points represent actual values for each individual mouse and zeros are plotted at the limit of detection. The dotted line indicates 10^4^ CFU/ml urine, which we use as a cutoff for persistent bacteriuria.
(TIF)

S5 Fig Bacterial strain-specific IgG can be detected in the serum of mice with a history of chronic cystitis. Serum from Adult Naive mice, mice with a history of chronic UTI89 or CFT073 cystitis (UTI89^HC^ and CFT073^HC^, respectively), and mice with a history of self-resolving UTI89 or CFT073 cystitis (UTI89^HR^ and CFT073^HR^, respectively) was used in an enzyme-linked immunosorbent assay (ELISA) with bacterial lysate from UTI89 (**A**) or CFT073 (**B**) as the coating antigen. The secondary antibody was HRP-conjugated goat-anti mouse IgG and the absorbance at 650 nm is shown. Samples were tested in duplicate and representative data from N = 3 experiments is shown.
(TIF)

S6 Fig Flow cytometry of splenic single cell suspensions was used to verify T cell depletion. Mice were infected with CFT073 and given weekly doses of 500 μg α-CD4 and 500 μg α-CD8, or 1 mg isotype control. At 28 dpi, mice with chronic cystitis were humanely sacrificed and spleens were harvested (“isotype (4 wk)” and “anti-CD4/8 (4 wk)”); other mice were treated with antibiotics as described above. Four weeks after the initiation of antibiotics, mice with a history of chronic cystitis were humanely sacrificed and spleens were harvested (“isotype (8 wk)” and “anti-CD4/8 (8 wk)”). As an additional control, spleens were obtained from C3H/HeN mice with no infection or antibody treatment (“no treatment”). After live lymphocyte gating, cell phenotypes were determined as follows: (**A**) CD3, CD4 and CD8a differentiate populations of T cells from CD19^+^ B cells. (**B**) CD11b^+^, Ly6G^+^ are neutrophils, CD11b^+^, Ly6C^+^ are monocytes, and F4/80^+^ cells are macrophages.
(TIF)

S7 Fig Depletion of CD4^+^ or CD8^+^ T cells did not cause a statistically significant increase in chronic CFT073 cystitis or impact the incidence of recurrent chronic CFT073 cystitis. Mice were treated with 500 μg α-CD4 or α-CD8, or isotype control (IgG2b), 24 hours prior to infection with 10^8^ CFU CFT073 and weekly thereafter. (**A**) Urines were collected to assess the infection outcome over four weeks. Dashed lines indicate our previously established cutoff for high-titer persistent bacteriuria, 10^4^ CFU/ml urine. Samples were unavailable for two isotype-treated mice at 1 dpi. (**B**) The overall incidence of chronic cystitis did not vary significantly among the groups. (**C**) Shown are the percentages of mice with persistent high-titer CFT073 bacteriuria (>10^4^ CFU/ml urine) over time. (**D and E**) Four weeks post infection, mice received antibiotics, and four weeks thereafter, mice that had developed chronic cystitis during the initial infection (CFT073^HC^) were challenged with 10^7^ CFU CFT073 and monitored over four weeks for the development of recurrent chronic cystitis. No additional antibody depletions were performed. (**D**) Urines were collected to monitor infection outcomes during the four week challenge period. Dashed lines indicate our previously established cutoff for high-titer persistent bacteriuria, 10^4^ CFU/ml urine. (**E**) Shown is the incidence of recurrent chronic cystitis during the challenge infection. One isotype-treated mouse had persistent bacteriuria but bladder titer <10^4^. Data are from two experiments. Data points represent actual values for each individual mouse, zeros are plotted at the limit of detection, and bars indicate median values; # denotes the number of mice per group.
(TIF)

## References

[1] Foxman B (2002) Epidemiology of urinary tract infections: incidence, morbidity, and economic costs. Am J Med 113 Suppl 1A: 5S–13S. 10.1016/s0002-9343(02)01054-9 12113866

[2] O’Brien VP, Hannan TJ, Nielsen HV, Hultgren SJ (2013) Drug and Vaccine Development for the Treatment and Prevention of Urinary Tract Infections Urinary Tract Infections: Molecular Pathogenesis and Clinical Management 2nd Edition. 10.1128/microbiolspec.UTI-0013-2012 PMC4887100 26999391

[3] Gilbert NM, O’Brien VP, Hultgren S, Macones G, Lewis WG, et al (2013) Urinary Tract Infection as a Preventable Cause of Pregnancy Complications: Opportunities, Challenges, and a Global Call to Action. Glob Adv Health Med 2: 11. 10.7453/gahmj.2013.061 PMC3833562 24416696

[4] Griebling T (2007) Urinary Tract Infections in Women Urologic Diseases in America.

[5] Mabeck CE (1972) Treatment of uncomplicated urinary tract infection in non-pregnant women. Postgrad Med J 48: 69–75. 4552445 PMC2495172

[6] Ferry SA, Holm SE, Stenlund H, Lundholm R, Monsen TJ (2004) The natural course of uncomplicated lower urinary tract infection in women illustrated by a randomized placebo controlled study. Scand J Infect Dis 36: 296–301. 15198188 10.1080/00365540410019642

[7] Christiaens TC, De Meyere M, Verschraegen G, Peersman W, Heytens S, et al (2002) Randomised controlled trial of nitrofurantoin versus placebo in the treatment of uncomplicated urinary tract infection in adult women. Br J Gen Pract 52: 729–734. 12236276 PMC1314413

[8] Bleidorn J, Gagyor I, Kochen MM, Wegscheider K, Hummers-Pradier E (2010) Symptomatic treatment (ibuprofen) or antibiotics (ciprofloxacin) for uncomplicated urinary tract infection?—results of a randomized controlled pilot trial. BMC Med 8: 30 10.1186/1741-7015-8-30 20504298 10.1186/1741-7015-8-30 PMC2890534

[9] Silverman JA, Schreiber HLt, Hooton TM, Hultgren SJ (2013) From physiology to pharmacy: developments in the pathogenesis and treatment of recurrent urinary tract infections. Curr Urol Rep 14: 448–456. 10.1007/s11934-013-0354-5 23832844 10.1007/s11934-013-0354-5 PMC3797163

[10] Godaly G, Ambite I, Svanborg C (2015) Innate immunity and genetic determinants of urinary tract infection susceptibility. Curr Opin Infect Dis 28: 88–96. 10.1097/QCO.0000000000000127 25539411 10.1097/QCO.0000000000000127 PMC4286230

[11] Hayes BW, Abraham SN (2016) Innate Immune Responses to Bladder Infection. Microbiol Spectr 4. 10.1128/microbiolspec.UTI-0024-2016 PMC5242417 28084200

[12] Thumbikat P, Waltenbaugh C, Schaeffer AJ, Klumpp DJ (2006) Antigen-specific responses accelerate bacterial clearance in the bladder. J Immunol 176: 3080–3086. 16493067 10.4049/jimmunol.176.5.3080

[13] Mora-Bau G, Platt AM, van Rooijen N, Randolph GJ, Albert ML, et al (2015) Macrophages Subvert Adaptive Immunity to Urinary Tract Infection. PLoS Pathog 11: e1005044 10.1371/journal.ppat.1005044 26182347 10.1371/journal.ppat.1005044 PMC4504509

[14] Hannan TJ, Mysorekar IU, Hung CS, Isaacson-Schmid ML, Hultgren SJ (2010) Early severe inflammatory responses to uropathogenic E. coli predispose to chronic and recurrent urinary tract infection. PLoS Pathog 6: e1001042 10.1371/journal.ppat.1001042 20811584 10.1371/journal.ppat.1001042 PMC2930321

[15] O’Brien VP, Hannan TJ, Yu L, Livny J, Roberson ED, et al (2016) A mucosal imprint left by prior Escherichia coli bladder infection sensitizes to recurrent disease. Nat Microbiol 2: 16196 10.1038/nmicrobiol.2016.196 27798558 10.1038/nmicrobiol.2016.196 PMC5308540

[16] Langermann S, Palaszynski S, Barnhart M, Auguste G, Pinkner JS, et al (1997) Prevention of mucosal Escherichia coli infection by FimH-adhesin-based systemic vaccination. Science 276: 607–611. 9110982 10.1126/science.276.5312.607

[17] Hannan TJ, Roberts PL, Riehl TE, van der Post S, Binkley JM, et al (2014) Inhibition of Cyclooxygenase-2 Prevents Chronic and Recurrent Cystitis. EBioMedicine 1: 46–57. 10.1016/j.ebiom.2014.10.011 26125048 10.1016/j.ebiom.2014.10.011 PMC4457352

[18] Mulvey MA, Schilling JD, Hultgren SJ (2001) Establishment of a persistent Escherichia coli reservoir during the acute phase of a bladder infection. Infect Immun 69: 4572–4579. 10.1128/IAI.69.7.4572-4579.2001 11402001 10.1128/IAI.69.7.4572-4579.2001 PMC98534

[19] Mobley HL, Green DM, Trifillis AL, Johnson DE, Chippendale GR, et al (1990) Pyelonephritogenic Escherichia coli and killing of cultured human renal proximal tubular epithelial cells: role of hemolysin in some strains. Infect Immun 58: 1281–1289. 2182540 10.1128/iai.58.5.1281-1289.1990 PMC258621

[20] Wiles TJ, Kulesus RR, Mulvey MA (2008) Origins and virulence mechanisms of uropathogenic Escherichia coli. Exp Mol Pathol 85: 11–19. 10.1016/j.yexmp.2008.03.007 18482721 10.1016/j.yexmp.2008.03.007 PMC2595135

[21] Schwartz DJ, Kalas V, Pinkner JS, Chen SL, Spaulding CN, et al (2013) Positively selected FimH residues enhance virulence during urinary tract infection by altering FimH conformation. Proc Natl Acad Sci U S A 110: 15530–15537. 10.1073/pnas.1315203110 24003161 10.1073/pnas.1315203110 PMC3785778

[22] Lloyd AL, Mobley HLT (2011) Fitness Islands in Uropathogenic Escherichia coli Population Genetics of Bacteria: A Tribute to Thomas S Whittam: 157–179.

[23] Mulvey MA, Lopez-Boado YS, Wilson CL, Roth R, Parks WC, et al (1998) Induction and evasion of host defenses by type 1-piliated uropathogenic Escherichia coli. Science 282: 1494–1497. 9822381 10.1126/science.282.5393.1494

[24] Hryckowian AJ, Baisa GA, Schwartz KJ, Welch RA (2015) dsdA Does Not Affect Colonization of the Murine Urinary Tract by Escherichia coli CFT073. PLoS One 10: e0138121 10.1371/journal.pone.0138121 26366567 10.1371/journal.pone.0138121 PMC4569052

[25] Kline KA, Schwartz DJ, Gilbert NM, Lewis AL (2014) Impact of host age and parity on susceptibility to severe urinary tract infection in a murine model. PLoS One 9: e97798 10.1371/journal.pone.0097798 24835885 10.1371/journal.pone.0097798 PMC4024022

[26] Nauciel C, Espinasse-Maes F (1992) Role of gamma interferon and tumor necrosis factor alpha in resistance to Salmonella typhimurium infection. Infect Immun 60: 450–454. 1730475 10.1128/iai.60.2.450-454.1992 PMC257648

[27] Parent MA, Berggren KN, Kummer LW, Wilhelm LB, Szaba FM, et al (2005) Cell-mediated protection against pulmonary Yersinia pestis infection. Infect Immun 73: 7304–7310. 10.1128/IAI.73.11.7304-7310.2005 16239527 10.1128/IAI.73.11.7304-7310.2005 PMC1273885

[28] Mastroeni P, Villarreal-Ramos B, Hormaeche CE (1992) Role of T cells, TNF alpha and IFN gamma in recall of immunity to oral challenge with virulent salmonellae in mice vaccinated with live attenuated aro- Salmonella vaccines. Microb Pathog 13: 477–491. 1363824 10.1016/0882-4010(92)90014-f

[29] Yee D, Rhinehart-Jones TR, Elkins KL (1996) Loss of either CD4+ or CD8+ T cells does not affect the magnitude of protective immunity to an intracellular pathogen, Francisella tularensis strain LVS. J Immunol 157: 5042–5048. 8943413

[30] Foxman B (2010) The epidemiology of urinary tract infection. Nat Rev Urol 7: 653–660. 10.1038/nrurol.2010.190 21139641 10.1038/nrurol.2010.190

[31] Sela U, Euler CW, Correa da Rosa J, Fischetti VA (2018) Strains of bacterial species induce a greatly varied acute adaptive immune response: The contribution of the accessory genome. PLoS Pathog 14: e1006726 10.1371/journal.ppat.1006726 29324905 10.1371/journal.ppat.1006726 PMC5764401

[32] Hopkins WJ, James LJ, Balish E, Uehling DT (1993) Congenital immunodeficiencies in mice increase susceptibility to urinary tract infection. J Urol 149: 922–925. 8455276 10.1016/s0022-5347(17)36260-2

[33] Kaas RS, Friis C, Ussery DW, Aarestrup FM (2012) Estimating variation within the genes and inferring the phylogeny of 186 sequenced diverse Escherichia coli genomes. BMC Genomics 13: 577 10.1186/1471-2164-13-577 23114024 10.1186/1471-2164-13-577 PMC3575317

[34] Schreiber HLt, Conover MS, Chou WC, Hibbing ME, Manson AL, et al (2017) Bacterial virulence phenotypes of Escherichia coli and host susceptibility determine risk for urinary tract infections. Sci Transl Med 9. 10.1126/scitranslmed.aaf1283 PMC5653229 28330863

[35] Kim KS (2003) Pathogenesis of bacterial meningitis: from bacteraemia to neuronal injury. Nat Rev Neurosci 4: 376–385. 10.1038/nrn1103 12728265 10.1038/nrn1103

[36] Hjelm EM (1984) Local cellular immune response in ascending urinary tract infection: occurrence of T-cells, immunoglobulin-producing cells, and Ia-expressing cells in rat urinary tract tissue. Infect Immun 44: 627–632. 6373612 10.1128/iai.44.3.627-632.1984 PMC263649

[37] Cowley SC, Hamilton E, Frelinger JA, Su J, Forman J, et al (2005) CD4-CD8- T cells control intracellular bacterial infections both in vitro and in vivo. J Exp Med 202: 309–319. 10.1084/jem.20050569 16027239 10.1084/jem.20050569 PMC2212999

[38] Cowley SC, Meierovics AI, Frelinger JA, Iwakura Y, Elkins KL (2010) Lung CD4-CD8- double-negative T cells are prominent producers of IL-17A and IFN-gamma during primary respiratory murine infection with Francisella tularensis live vaccine strain. J Immunol 184: 5791–5801. 10.4049/jimmunol.1000362 20393138 10.4049/jimmunol.1000362

[39] Cowley SC, Elkins KL (2003) Multiple T cell subsets control Francisella tularensis LVS intracellular growth without stimulation through macrophage interferon gamma receptors. J Exp Med 198: 379–389. 10.1084/jem.20030687 12885873 10.1084/jem.20030687 PMC2194083

[40] Jones-Carson J, Balish E, Uehling DT (1999) Susceptibility of immunodeficient gene-knockout mice to urinary tract infection. J Urol 161: 338–341. 10037434

[41] Tubo NJ, Jenkins MK (2014) CD4+ T Cells: guardians of the phagosome. Clin Microbiol Rev 27: 200–213. 10.1128/CMR.00097-13 24696433 10.1128/CMR.00097-13 PMC3993097

[42] Chan CY, St John AL, Abraham SN (2013) Mast cell interleukin-10 drives localized tolerance in chronic bladder infection. Immunity 38: 349–359. 10.1016/j.immuni.2012.10.019 23415912 10.1016/j.immuni.2012.10.019 PMC3647685

[43] Sivick KE, Schaller MA, Smith SN, Mobley HL (2010) The innate immune response to uropathogenic Escherichia coli involves IL-17A in a murine model of urinary tract infection. J Immunol 184: 2065–2075. 10.4049/jimmunol.0902386 20083670 10.4049/jimmunol.0902386 PMC2821792

[44] McGann P, Snesrud E, Maybank R, Corey B, Ong AC, et al (2016) Escherichia coli Harboring mcr-1 and blaCTX-M on a Novel IncF Plasmid: First Report of mcr-1 in the United States. Antimicrob Agents Chemother 60: 4420–4421. 10.1128/AAC.01103-16 27230792 10.1128/AAC.01103-16 PMC4914657

[45] Hooton TM, Scholes D, Hughes JP, Winter C, Roberts PL, et al (1996) A prospective study of risk factors for symptomatic urinary tract infection in young women. N Engl J Med 335: 468–474. 10.1056/NEJM199608153350703 8672152 10.1056/NEJM199608153350703

[46] O’Brien VP, Hannan TJ, Nielsen HV, Hultgren SJ (2016) Drug and Vaccine Development for the Treatment and Prevention of Urinary Tract Infections. Microbiol Spectr 4. 10.1128/microbiolspec.UTI-0013-2012 PMC4887100 26999391

[47] Wright KJ, Seed PC, Hultgren SJ (2005) Uropathogenic Escherichia coli flagella aid in efficient urinary tract colonization. Infect Immun 73: 7657–7668. 10.1128/IAI.73.11.7657-7668.2005 16239570 10.1128/IAI.73.11.7657-7668.2005 PMC1273872

[48] Hung CS, Dodson KW, Hultgren SJ (2009) A murine model of urinary tract infection. Nat Protoc 4: 1230–1243. 10.1038/nprot.2009.116 19644462 10.1038/nprot.2009.116 PMC2963178

[49] Walker JN, Flores-Mireles AL, Pinkner CL, Schreiber HLt, Joens MS, et al (2017) Catheterization alters bladder ecology to potentiate Staphylococcus aureus infection of the urinary tract. Proc Natl Acad Sci U S A 114: E8721–E8730. 10.1073/pnas.1707572114 28973850 10.1073/pnas.1707572114 PMC5642702

[50] Gilbert NM, O’Brien VP, Lewis AL (2017) Transient microbiota exposures activate dormant Escherichia coli infection in the bladder and drive severe outcomes of recurrent disease. PLoS Pathog 13: e1006238 10.1371/journal.ppat.1006238 28358889 10.1371/journal.ppat.1006238 PMC5373645

