# Supplementary figures and images for: Host restriction of Escherichia coli recurrent urinary tract infection occurs in a bacterial strain-specific manner
Source: PLoS Pathog. 2018 Dec 13;14(12):e1007457. doi: 10.1371/journal.ppat.1007457 (PMC6292575; doi:10.1371/journal.ppat.1007457)

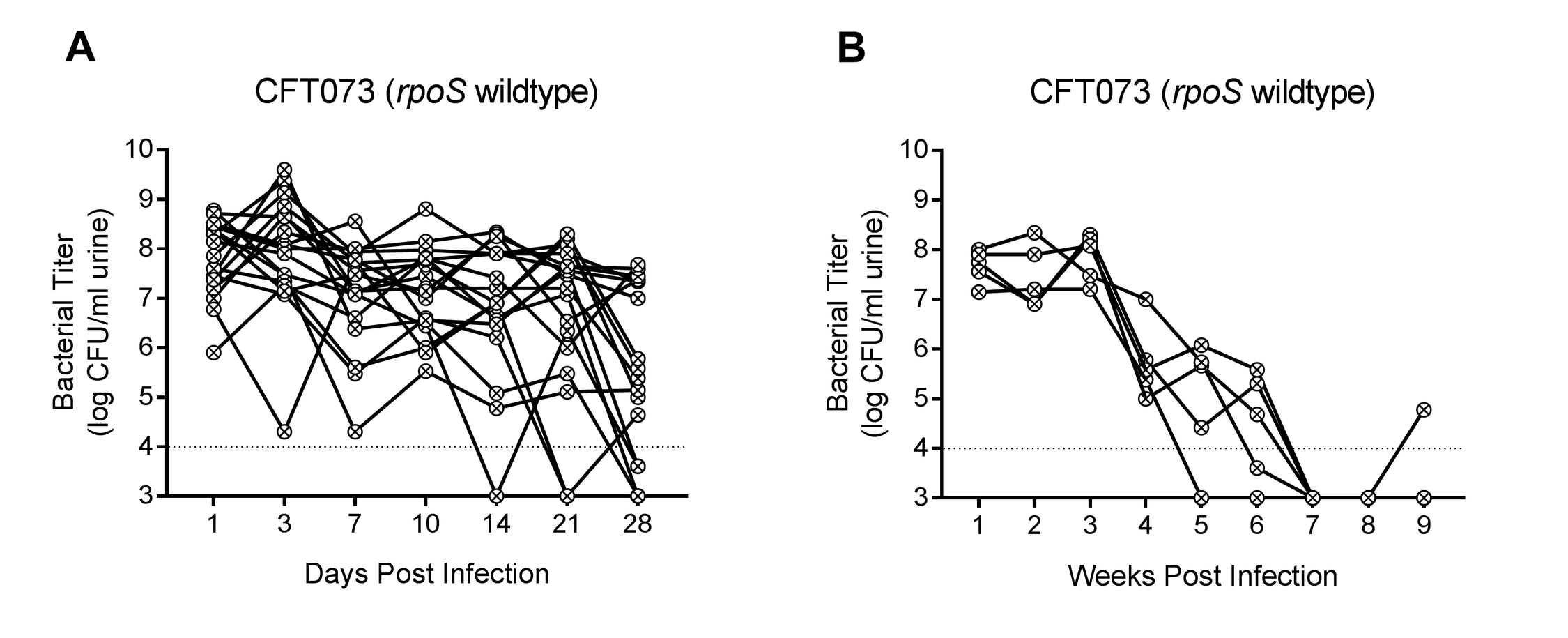

Supplement: S1 Fig — (A) Mice were infected with a strain of CFT073 with a wild-type rpoS allele, verified by sequencing using the primers in [24], and followed over four weeks. Data are from two experiments; data points represent actual values for each individual mouse and zeros are plotted at the limit of detection. (B) A subset of mice (not depicted in panel A) that were still chronically infected at four weeks were followed for an additional five weeks, during which time all mice resolved the infection. (TIF) [file ppat.1007457.s001.tif]

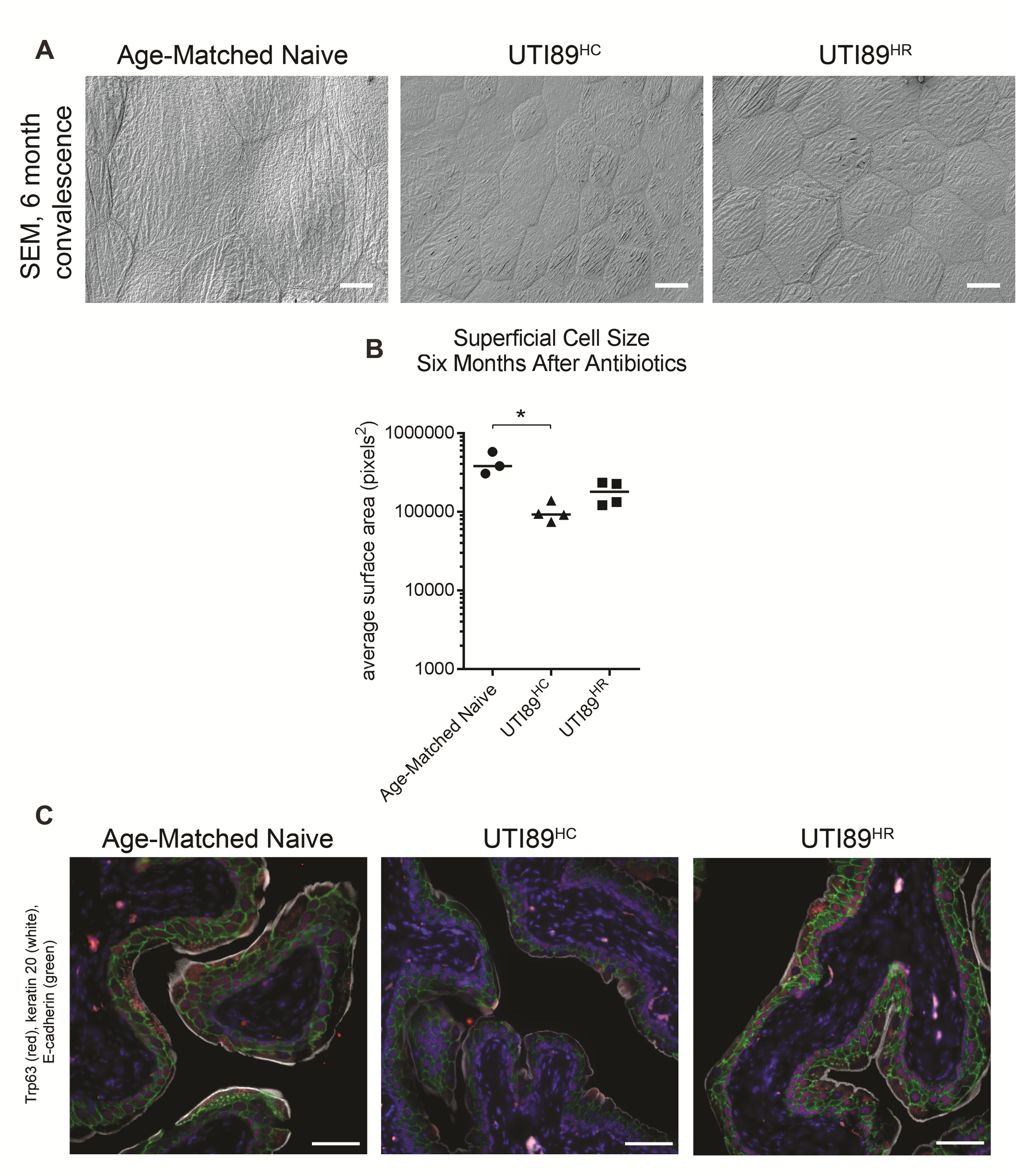

Supplement: S2 Fig — Mice were initially mock-infected (“Age-Matched Naive”), or infected with UTI89. Antibiotics were initiated at four wpi, and mice were allowed to convalesce for six months. (A and B) Scanning electron microscopy (SEM) was used to assess urothelial superficial cell size in the Naive mice vs. mice that initially had a chronic UTI89 infection (UTI89HC) or initially resolved their UTI89 infection (UTI89HR). (A) Representative images from N = 2 replicates with a total of n = 3–4 mice per group are shown. Scale bars, 25 μm. (B) Superficial cells from N = 2 replicates with a total of n = 3–4 mice per group were measured in ImageJ and the average surface area (pixels2) was calculated. Data points represent the average of all measurements for a given mouse. * P < 0.05, Kruskal-Wallis test with Dunn’s multiple test correction. (C) Immunofluorescence microscopy was performed on paraffin-embedded bladder sections from N = 2 staining experiments with bladder sections from n = 3 Adult Naive mice, N = 5 UTI89HC mice, and N = 4 UTI89HR mice; representative images are shown. Keratin 20 is shown in white, E-cadherin in green, Trp63 in red and nuclei in blue. Scale bars, 50 μm. (TIF) [file ppat.1007457.s002.tif]

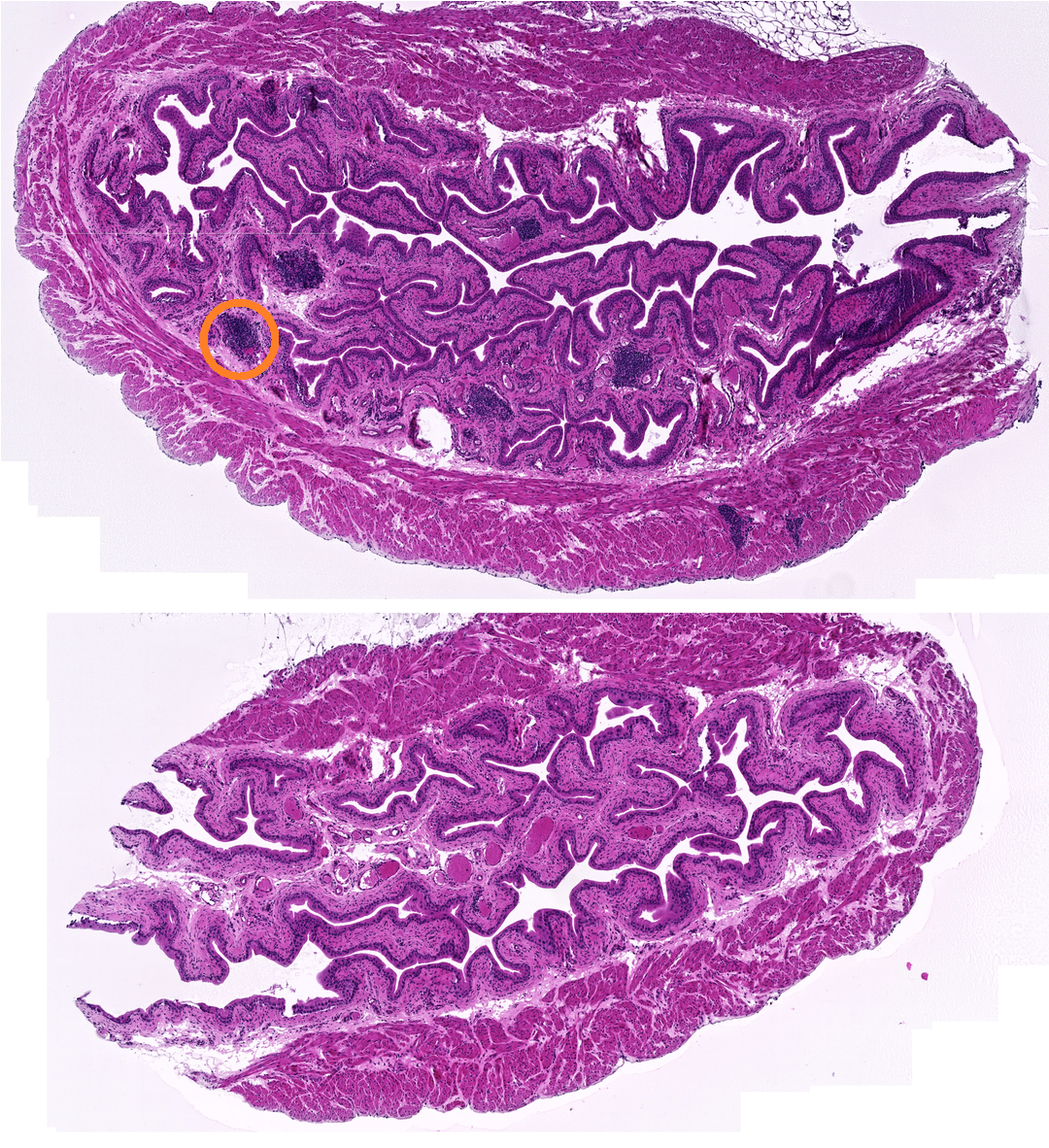

Supplement: S3 Fig — We previously observed that chronic UTI89 infection results in lymphoid follicles that persist after antibiotic therapy [14]. Here bladders were harvested from CFT073HC mice four weeks after antibiotic therapy. Histological examination of hematoxylin & eosin-stained sections shows evidence of lymphoid follicles in the lamina propria of CFT073HC mice (top; example circled in orange). Lymphoid follicles were not observed in mice that spontaneously resolved a CFT073 infection (bottom). Representative images from N = 2 replicates with a total of n = 4 mice are shown. (TIF) [file ppat.1007457.s003.tif]

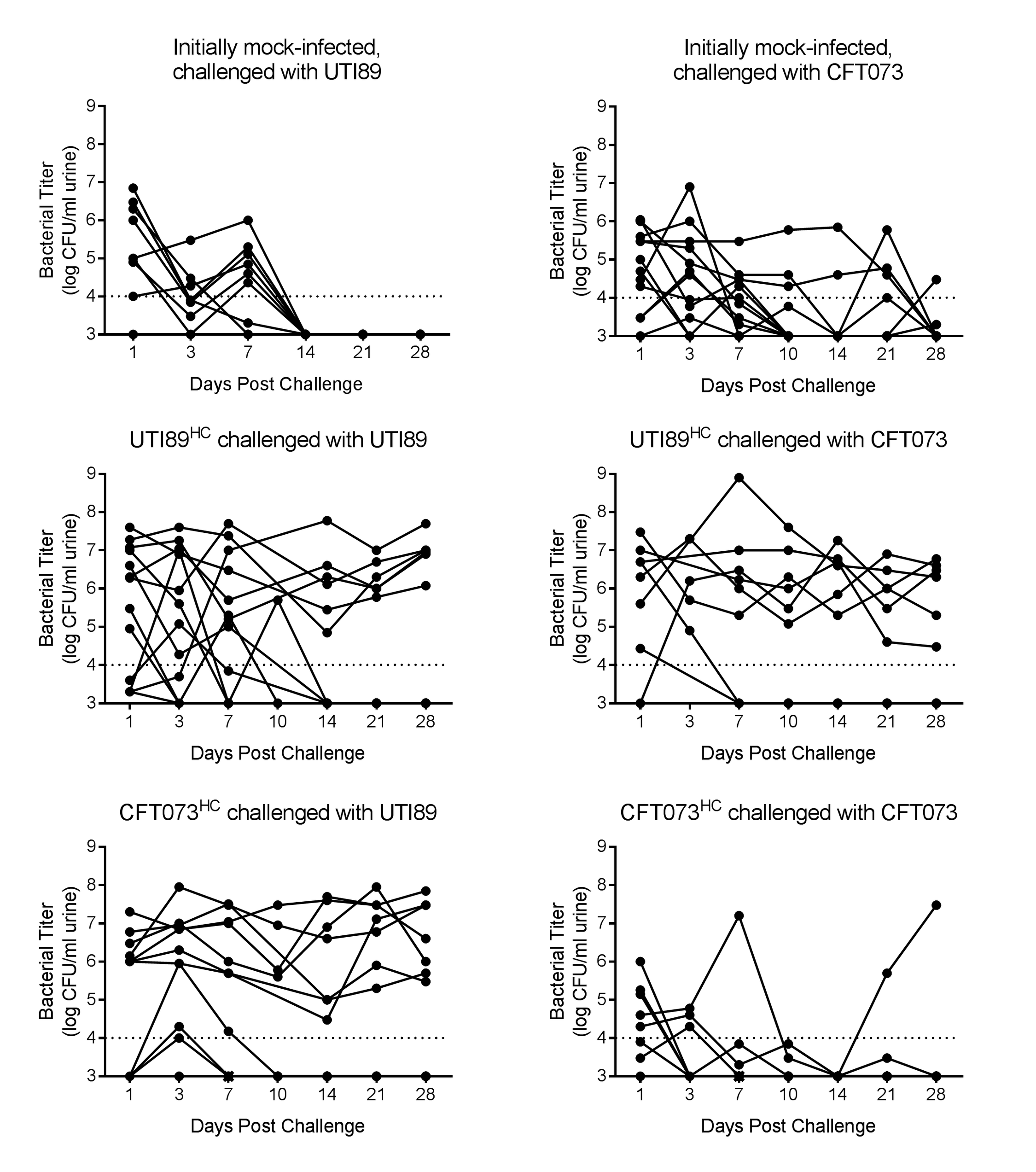

Supplement: S4 Fig — Shown are the urine titers during challenge infection for mice in Fig 5B and 5C. Mice were initially infected with 108 CFU UTI89 or CFT073, or mock-infected, and monitored over four weeks, at which time antibiotic therapy was initiated. Four weeks after antibiotics, mice were challenged with 107 CFU CFT073 or UTI89. We note the instance of two “late resolvers” in the initially mock-infected (Adult Naive) mice challenged with CFT073 (see Fig 2G). Data are combined from two to three independent experiments; data points represent actual values for each individual mouse and zeros are plotted at the limit of detection. The dotted line indicates 104 CFU/ml urine, which we use as a cutoff for persistent bacteriuria. (TIF) [file ppat.1007457.s004.tif]

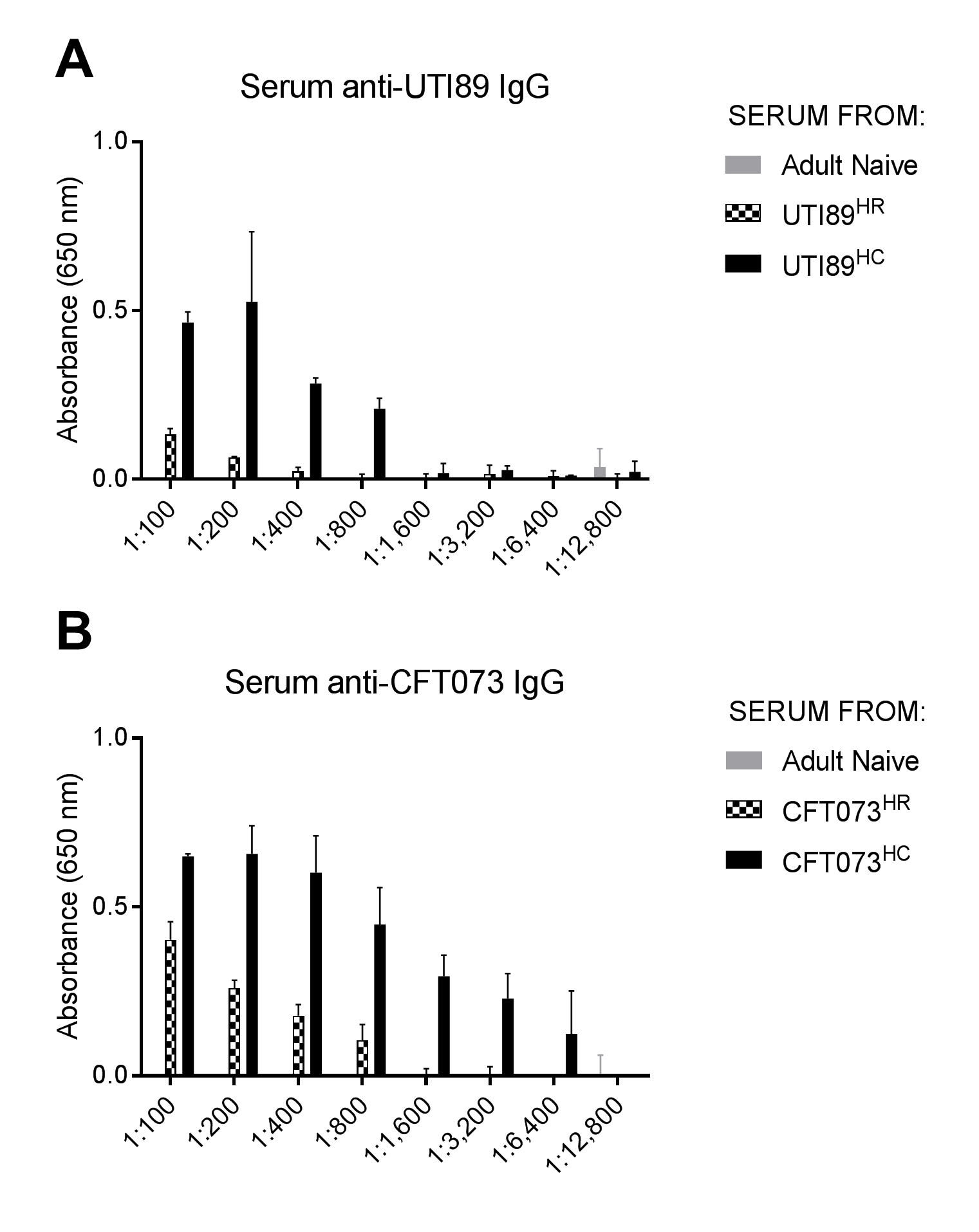

Supplement: S5 Fig — Serum from Adult Naive mice, mice with a history of chronic UTI89 or CFT073 cystitis (UTI89HC and CFT073HC, respectively), and mice with a history of self-resolving UTI89 or CFT073 cystitis (UTI89HR and CFT073HR, respectively) was used in an enzyme-linked immunosorbent assay (ELISA) with bacterial lysate from UTI89 (A) or CFT073 (B) as the coating antigen. The secondary antibody was HRP-conjugated goat-anti mouse IgG and the absorbance at 650 nm is shown. Samples were tested in duplicate and representative data from N = 3 experiments is shown. (TIF) [file ppat.1007457.s005.tif]

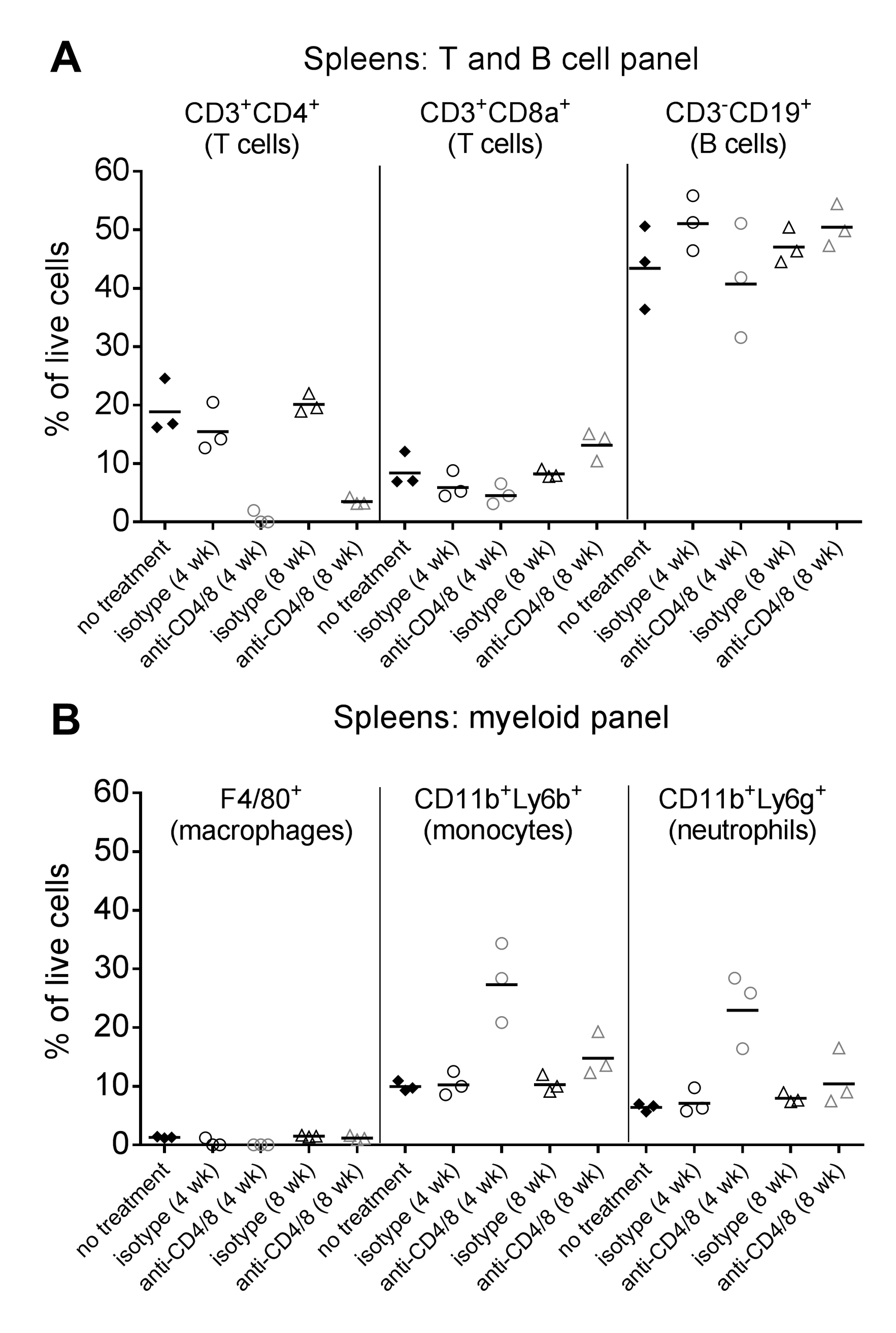

Supplement: S6 Fig — Mice were infected with CFT073 and given weekly doses of 500 μg α-CD4 and 500 μg α-CD8, or 1 mg isotype control. At 28 dpi, mice with chronic cystitis were humanely sacrificed and spleens were harvested (“isotype (4 wk)” and “anti-CD4/8 (4 wk)”); other mice were treated with antibiotics as described above. Four weeks after the initiation of antibiotics, mice with a history of chronic cystitis were humanely sacrificed and spleens were harvested (“isotype (8 wk)” and “anti-CD4/8 (8 wk)”). As an additional control, spleens were obtained from C3H/HeN mice with no infection or antibody treatment (“no treatment”). After live lymphocyte gating, cell phenotypes were determined as follows: (A) CD3, CD4 and CD8a differentiate populations of T cells from CD19+ B cells. (B) CD11b+, Ly6G+ are neutrophils, CD11b+, Ly6C+ are monocytes, and F4/80+ cells are macrophages. (TIF) [file ppat.1007457.s006.tif]

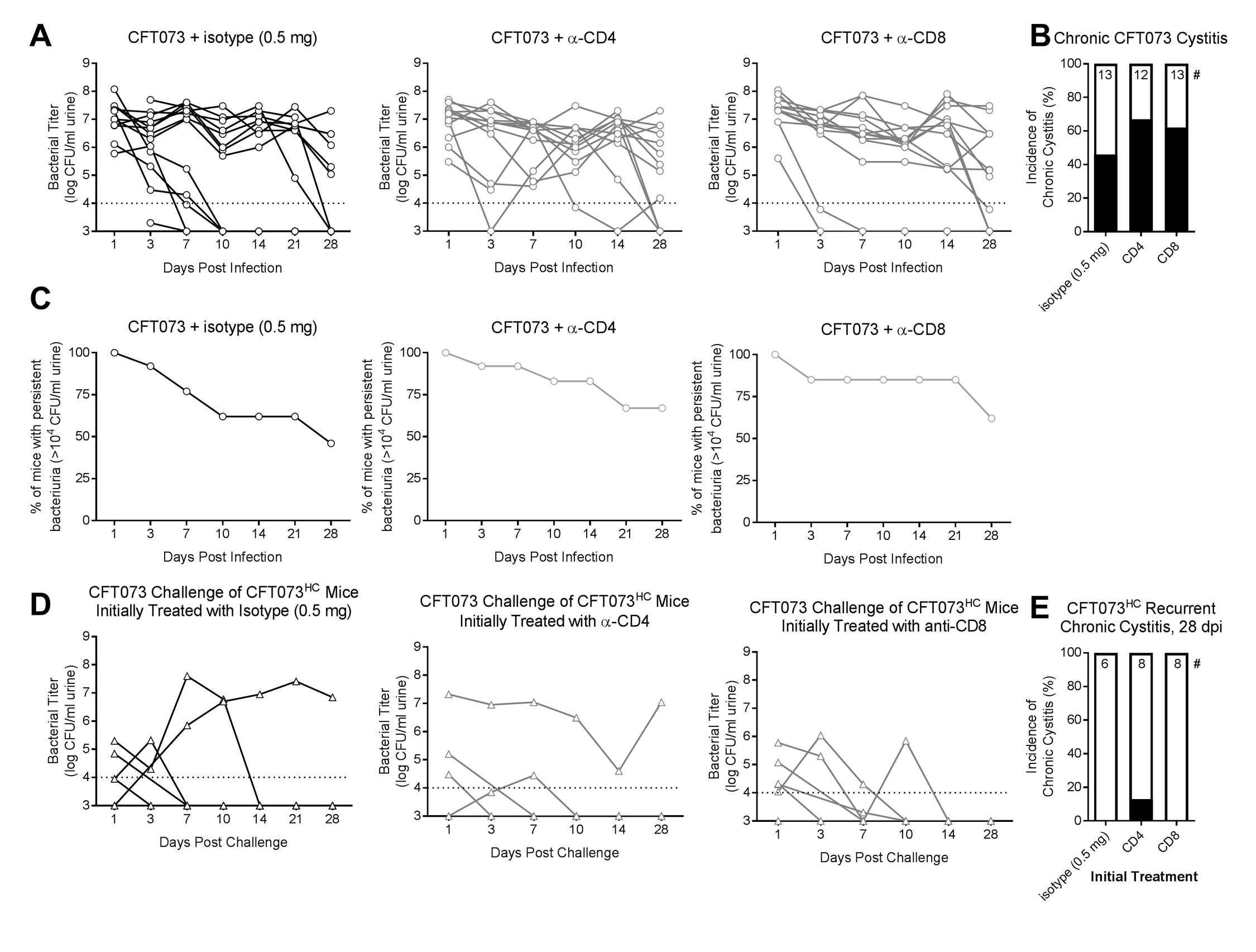

Supplement: S7 Fig — Mice were treated with 500 μg α-CD4 or α-CD8, or isotype control (IgG2b), 24 hours prior to infection with 108 CFU CFT073 and weekly thereafter. (A) Urines were collected to assess the infection outcome over four weeks. Dashed lines indicate our previously established cutoff for high-titer persistent bacteriuria, 104 CFU/ml urine. Samples were unavailable for two isotype-treated mice at 1 dpi. (B) The overall incidence of chronic cystitis did not vary significantly among the groups. (C) Shown are the percentages of mice with persistent high-titer CFT073 bacteriuria (>104 CFU/ml urine) over time. (D and E) Four weeks post infection, mice received antibiotics, and four weeks thereafter, mice that had developed chronic cystitis during the initial infection (CFT073HC) were challenged with 107 CFU CFT073 and monitored over four weeks for the development of recurrent chronic cystitis. No additional antibody depletions were performed. (D) Urines were collected to monitor infection outcomes during the four week challenge period. Dashed lines indicate our previously established cutoff for high-titer persistent bacteriuria, 104 CFU/ml urine. (E) Shown is the incidence of recurrent chronic cystitis during the challenge infection. One isotype-treated mouse had persistent bacteriuria but bladder titer <104. Data are from two experiments. Data points represent actual values for each individual mouse, zeros are plotted at the limit of detection, and bars indicate median values; # denotes the number of mice per group. (TIF) [file ppat.1007457.s007.tif]
